# Dnd1-mediated epigenetic control of teratoma formation in mouse

**DOI:** 10.1242/bio.032318

**Published:** 2018-01-15

**Authors:** Wei Gu, Kentaro Mochizuki, Kei Otsuka, Ryohei Hamada, Asuka Takehara, Yasuhisa Matsui

**Affiliations:** 1 Cell Resource Center for Biomedical Research, Institute of Development, Aging and Cancer (IDAC), Tohoku University, 4-1 Seiryo-machi, Aoba-ku, Sendai, Miyagi 980-8575, Japan; 2 Laboratory of Germ Cell Development, Graduate School of Life Sciences, Tohoku University, 2-1-1 Katahira, Aoba-ku, Sendai, Miyagi 980-8577, Japan; 3 The Japan Agency for Medical Research and Development-Core Research for Evolutional Science and Technology (AMED-CREST), Tokyo 100-0004, Japan; 4 Center for Regulatory Epigenome and Diseases, Tohoku University School of Medicine, 2-1 Seiryo-machi, Aoba-ku, Sendai, Miyagi 980-8575, Japan

**Keywords:** Primordial germ cell, Teratoma, Histone methylation, Dnd1

## Abstract

Spontaneous testicular teratoma develops from primordial germ cells (PGCs) in embryos; however, the molecular mechanisms underlying teratoma formation are not fully understood. Mutation of the dead-end 1 (*Dnd1*) gene, which encodes an RNA-binding protein, drastically enhances teratoma formation in the 129/Sv mouse strain. To elucidate the mechanism of *Dnd1* mutation-induced teratoma formation, we focused on histone H3 lysine 27 (H3K27) trimethylation (me3), and found that the levels of H3K27me3 and its responsible methyltransferase, enhancer of zeste homolog 2 (Ezh2), were decreased in the teratoma-forming cells of *Dnd1* mutant embryos. We also showed that Dnd1 suppressed miR-26a-mediated inhibition of Ezh2 expression, and that Dnd1 deficiency resulted in decreased H3K27me3 of a cell-cycle regulator gene, *Ccnd1*. In addition, Ezh2 expression or Ccnd1 deficiency repressed the reprogramming of PGCs into pluripotent stem cells, which mimicked the conversion of embryonic germ cells into teratoma-forming cells. These results revealed an epigenetic molecular linkage between Dnd1 and the suppression of testicular teratoma formation.

## Abstract (precis)

**Summary:** Teratoma development from primordial germ cells is repressed by downregulation of Ccnd1 via histone H3 lysine 27 methylation controlled by Dnd1-mediated maintenance of Ezh2.

## INTRODUCTION

Teratoma originates from primordial germ cells (PGCs), which are undifferentiated germ cells in embryos, and contains differentiated cells of all three germ layers as well as pluripotent stem cells. During teratoma formation, embryonic germ cells initially form a cluster of early embryonic cells that are likely pluripotent. In humans, 3% of adult and 38% of childhood testicular cancers are teratomas (Bustamante-Marín et al., 2013). The mechanisms of teratoma formation have been studied in mice. Testicular teratoma is often found in the 129/Sv mouse strain, and a *Ter* mutation in the 129/Sv background significantly enhances the incidence of teratoma (Stevens, 1973). Some teratomas are also found in mouse ovary. A deficiency of Retinoblastoma 1 (Rb1) or Forkhead box O3a (Foxo3a) has been shown to cause oocyte abnormality and ovarian teratoma. Although the mechanisms behind these remain unclear, the dysregulation of follicle growth due to Rb1 deficiency and impaired Foxo3a-directed apoptosis are likely associated with teratoma formation (Yang et al., 2015; Youngson et al., 2011). In addition, the LT/Sv mouse strain exhibits a high frequency of spontaneous ovarian teratoma (Eppig et al., 1996).

The conditional loss of phosphatase and tensin homolog (*Pten*) in PGCs leads to both testicular and ovarian teratomas, perhaps owing to the activation of the phosphoinositide 3-kinase (PI3K)/RAC-alpha serine/threonine-protein kinase (Akt) pathway that is suppressed by Pten (Kimura et al., 2003; Ramaswamy et al., 1999). Akt activation results in the inactivation of p53 and the subsequent suppression of apoptosis in PGCs. A *Ter* mutation normally causes teratoma only in the 129/Sv genetic background, but a high incidence of teratoma, is also found in the C57BL/6J and 129/Sv mixed background when a mutation in an apoptotic gene, *Bax*, is additionally introduced (Cook et al., 2011). Taken together, these observations suggest that the suppression of apoptosis plays a role in teratoma formation.

In addition to the suppression of apoptosis, the abnormal stimulation of mitosis in PGCs is also involved in teratoma formation. The mitotic proliferation of PGCs is normally arrested by embryonic day (E) 14.5 in male mice (Western et al., 2008). In the testis of 129/Sv mice, loss of the transcription regulator Dmrt1, which binds to the promoter of *p19^Ink4d^*, encoding a negative regulator of the G1-S transition of the cell cycle, results in the downregulation of *p19^Ink4^* expression and causes a high incidence of teratoma (Krentz et al., 2009). The dead-end 1 (*Dnd1*) gene, which encodes an RNA-binding protein, was identified as the gene responsible for *Ter* mutants, and a point mutation that introduced a stop codon was found in the third exon of the *Dnd1* gene in *Ter* mutant mice (Youngren et al., 2005). The Dnd1 protein associates with uridine-rich regions in the 3′-untranslated region (3′-UTR) of target mRNAs, and protects the mRNAs from microRNA (miRNA)-mediated translation repression (Kedde et al., 2007). *p21^cip1^* and *p27^kip1^* mRNAs, which also encode negative regulators of the cell cycle, were identified as targets of Dnd1, and a deficiency of Dnd1 in *Ter* mutants resulted in downregulation of the p21^cip1^ and p27^Kip^ proteins (Cook et al., 2011). The above findings demonstrate that a number of molecules are involved in teratoma formation, and enhancement of the cell cycle and suppression of apoptosis are likely key events for the conversion of PGCs into teratoma.

Epigenetic regulation is involved in various changes of cellular status, including the differentiation and reprogramming of cells, and during PGC development, dynamic epigenetic changes occur. DNA methylation occurs at a high level in E6.5 epiblasts, but PGCs undergo global erasure of DNA methylation and gradually become hypomethylated after E9.5; by E13.5, only ∼10% of the genome is methylated (Seisenberger et al., 2012). PGCs also show decreasing levels of histone H3 lysine 9 di-methylation (H3K9me2), a repressive modification, from E8.0, and H3K9me2 is subsequently maintained at a low level until at least E18.5 in male mice (Seki et al., 2005; Deguchi et al., 2013). In the case of histone H3 lysine 27 tri-methylation (H3K27me3), which is another repressive modification, its level increases after E8.0 and is maintained at a high level until at least E12.5 (Seki et al., 2005). H3K27me3 is involved in the repression of somatic and meiotic genes in PGCs (Mu et al., 2014). PR/SET domain 14 *(Prdm14*) encodes a protein containing a PR/SET domain, a motif of histone methyltransferase, although its activity has not yet been determined. Deficiency of Prdm14 causes increased levels of H3K9me2 and decreased levels of H3K27me3 in PGCs; subsequently, the PGCs disappear by E12.5 (Yamaji et al., 2008). In addition, deficiency of ubiquitously transcribed tetratricopeptide repeat, X chromosome (UTX), a H3K27me3 demethylase, causes high levels of H3K27me3 at E10.5, as well as the failed expression of Nanog, Sal-like protein 4 (Sal4), octomer-binding transcription factor 4 (Oct4) and stage-specific embryonic antigen 1 (SSEA1) at E12.5 in PGCs (Mansour et al., 2012).

Due to the dynamic changes and the important roles of epigenetic modifications in PGC development and of the differential histone modification levels in PGCs and pluripotent stem cells, we hypothesized that abnormal changes in histone modification in *Dnd1*-mutant PGCs and later embryonic germ cells are involved in the initial steps of teratoma formation, i.e. the conversion of germ cells into pluripotent teratoma-forming cells. Our results showed a molecular linkage between Dnd1, its target, enhancer of zeste homolog 2 (Ezh2), as well as a target of Ezh2, cyclin D1 (Ccnd1), in the conversion of embryonic germ cells into teratoma-forming cells.

## RESULTS

### Mutation of *Dnd1* causes abnormal histone methylation in testicular teratoma-forming cells

Testicular teratoma develops from embryonic germ cells. In a previous study, teratoma-forming *Dnd1*-mutant germ cells in the 129Sv background were initially identified as morphologically abnormal cells showing a high nucleo-cytoplasmic ratio at as early as E14 (Rivers and Hamilton, 1986). However, the differences between teratoma-forming cells and embryonic germ cells are still not obvious at the molecular level. We were interested in the possible epigenetic differences between teratoma-forming cells and PGCs/embryonic germ cells, because the epigenetic status of pluripotent stem cells is different from that of PGCs. We focused on two repressive histone modifications, H3K27me3 and H3K9me2, because they show particularly characteristic changes and play crucial roles in embryonic germ cell development.

**Fig. 1.**
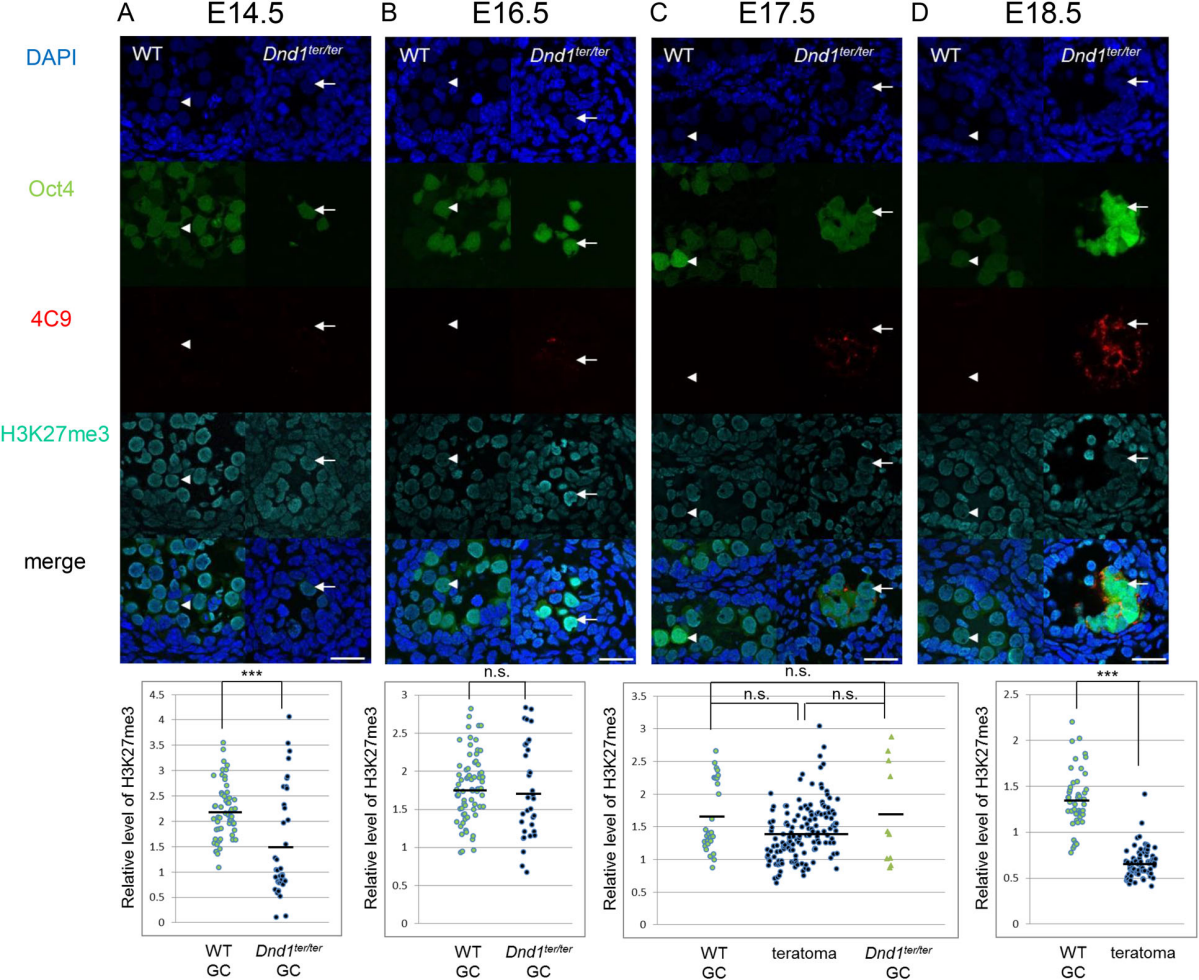
**H3K27me3 in the teratoma-forming cells and germ cells in *Dnd1^ter/ter^* testes and in wild-type/*Dnd1^ter/+^*** **(WT)** **testes at E14.5 to E18.5.** (A-D) The Oct4-ΔPE-GFP-positive germ cells (arrowheads in A,D) in WT testes showed higher H3K27me3 signals than the 4C9-positive teratoma-forming cells at E18.5 (arrows in D) and GFP-positive germ cells at E14.5 (arrows in A) in *Dnd1^ter/ter^* testes. The H3K27me3 signals were comparable between the teratoma-forming cells and germ cells in WT and *Dnd1^ter/ter^* testes at E16.5 and E17.5 (B,C). Results of the quantitative analysis of the H3K27me3 signal intensity in WT or *Dnd1^ter/ter^* germ cells (GC) and teratoma-forming cells (teratoma) relative to the surrounding somatic cells are shown at the bottom of the pictures. Comparisons of the somatic cells with germ cells and with teratoma-forming cells are shown in Fig. S2. The average signal intensity of 10 randomly selected somatic cells in each section was set as 1, and the signal intensity of each germ cell or teratoma-forming cell relative to the average value of the somatic cells in the same observed section was estimated. In total, three to five sections from three embryos of each genotype were observed. ***P<0.001; n.s., not significantly different. Scale bars: 25 μm.

In E18.5 testes, clusters of cells positive for the pluripotent cell marker 4C9 (Yoshinaga et al., 1991), green fluorescence protein (GFP) from the Oct4-ΔPE-GFP transgene (Fig. 1D), and endogenous Sox2 (Fig. S1A) were obvious in *Dnd1* mutants (*Dnd1^ter/ter^*), but in wild-type mice, all of the GFP-positive germ cells were 4C9 negative (Fig. 1D). We defined the 4C9- and Oct4-ΔPE-GFP-expressing cells in clusters as teratoma-forming cells in this study. We found that H3K27me3 was significantly decreased in the 4C9-positive teratoma-forming cells in *Dnd1^ter/ter^* testes when compared to the wild-type germ cells (Fig. 1D; Fig. S2). In contrast, H3K9me2 was higher in the *Dnd1*^ter/ter^ teratoma-forming cells than in the wild-type germ cells (Fig. S3A-D). In this study, we focused on H3K27me3 in the regulation of teratoma formation.

### Mutation of *Dnd1* causes the downregulation of Ezh2 in teratoma-forming cells

**Fig. 2.**
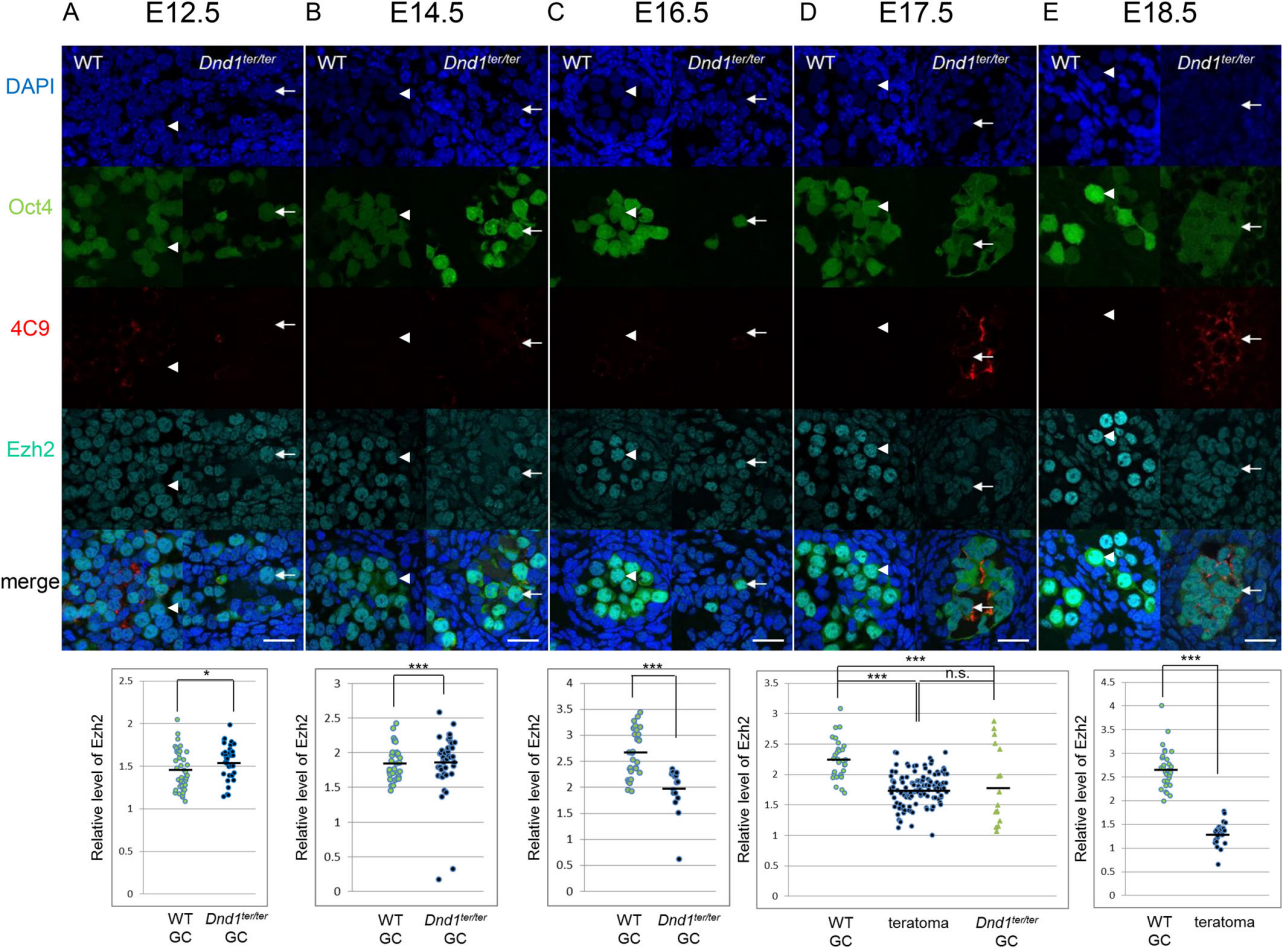
**Ezh2 expression in the teratoma-forming cells and germ cells in *Dnd1^ter/ter^* testes and in wild-type/*Dnd1^ter/+^* (WT) testes at E12.5 to E18.5.** (A-E) The Oct4-ΔPE-GFP-positive germ cells (arrowheads in C,D,E) in WT testes showed higher Ezh2 signals than the 4C9-positive teratoma-forming cells at E17.5 (arrows in D) and E18.5 (arrows in E) as well as the GFP-positive germ cells at E16.5 (arrows in C) and E17.5 (D) in *Dnd1^ter/ter^* testes. The GFP-positive germ cells in WT testes (arrowheads) showed slightly less Ezh2 signals than those in *Dnd1^ter/ter^* testes (arrows) at E12.5 (A) and E14.5 (B). Results of the quantitative analysis of the Ezh2 signal intensity in WT or *Dnd1^ter/ter^* germ cells (GC) and teratoma-forming cells (teratoma) relative to the surrounding somatic cells are shown at the bottom of the pictures. Comparisons of the somatic cells with germ cells and with teratoma-forming cells are shown in Fig. S4. The average signal intensity of 10 randomly selected somatic cells in each section was set as 1, and the signal intensity of each germ cell or teratoma-forming cell relative to the average value of the somatic cells in the same observed section was estimated. In total, three to five sections from three embryos of each genotype were observed. *P<0.05, ***P<0.001; n.s., not significantly different. Scale bars: 25 μm.

In mammalians, polycomb protein complex 2 (PRC2) catalyzes H3K27me3. Ezh2 and suppressor of zeste 12 (Suz12) are two core members of PRC2, and Ezh2 is responsible for catalyzing the methylation on H3K27. The Suz12-stabilizing methyltransferase activity of Ezh2 is also required for the functions of PRC2 (Cao et al., 2002; Cao and Zhang, 2004; Pasini et al., 2004). We investigated whether the decrease in H3K27me3 in *Dnd1^ter/ter^* germ cells was associated with the downregulation of Ezh2 and/or Suz12. In E18.5 testes, the expression of Ezh2 and Suz12 was significantly down- and upregulated, respectively, in the teratoma-forming cells in *Dnd1^ter/ter^* testes when compared to wild-type germ cells (Fig. 2E; Fig. S3E-H, Fig. S4). These results suggested that the downregulation of Ezh2 caused decreased H3K27me3 in the teratoma-forming cells in *Dnd1^ter/ter^* testes.

### H3K27me3 and the expression of Ezh2 in earlier *Dnd1^ter/ter^* testicular germ cells

We next examined earlier embryonic testes. In E17.5 *Dnd1^ter/ter^* testes, we found fewer and smaller clusters of 4C9-positive cells (Fig. 1C, Fig. 2D); in contrast, no 4C9-positive cell clusters were observed in the wild-type testes. At E16.5, faint 4C9 signals were occasionally observed in a few germ cells both in wild-type and *Dnd1^ter/ter^* testes, but they did not form the cell clusters that are characteristic of teratoma-forming cells (Fig. 1B, Fig. 2C). To further characterize the teratoma-forming cells, we tested for the embryonic germ cell markers Mvh (Toyooka et al., 2000) and TRA98 (Tanaka et al., 1997). In E18.5 *Dnd1^ter/ter^* testes, the expression of Mvh was negative (Fig. S5D). At E17.5, Mvh was undetectable or found at very low levels in the teratoma-forming cells (Fig. S5B,C), and a few scattered germ cells expressed Mvh at various intensities in *Dnd1^ter/ter^* testes (Fig. S5B). In contrast, TRA98 was expressed not only in wild-type germ cells, but also in *Dnd1^ter/ter^* germ cells as well as in teratoma-forming cell clusters expressing Oct4-ΔPE-GFP at E17.5 (Fig. S5B). At E16.5, Mvh was expressed both in wild-type and *Dnd1^ter/ter^* germ cells (Fig. S5A). These results indicated that Mvh is downregulated during the development of teratoma-forming cells from germ cells after E16.5. We also examined changes in the number of germ cells in wild-type and *Dnd1^ter/ter^* testes, and found that the number of Oct4-ΔPE-GFP-expressing germ cells largely decreased between E14.5 and E16.5 (Fig. S6). This suggested that the majority of germ cells are progressively lost during embryogenesis in *Dnd1^ter/ter^* testes, and the few remaining germ cells become Mvh-negative and begin to be converted into teratoma-forming cells.

We next investigated whether *Dnd1^ter/ter^* germ cells at earlier stages also show less H3K27me3 when compared to wild-type germ cells. The wild-type and *Dnd1^ter/ter^* germ cells showed similar levels of H3K27me3, which were higher than those in the surrounding somatic cells at E17.5 and E16.5. (Fig. 1B,C; Fig. S2). The teratoma-forming cells at E17.5 also showed similar expression levels when compared to the levels in germ cells. In addition, the expression of Ezh2 was downregulated in *Dnd1^ter/ter^* germ cells at E16.5 and E17.5, and in the teratoma-forming cells at E17.5, when compared to the levels in wild-type germ cells (Fig. 2C,D; Fig. S4). These results suggested that the Dnd1 deficiency-dependent downregulation of Ezh2 precedes the decrease in H3K27me3 in embryonic germ cells and teratoma-forming cells in *Dnd1^ter/ter^* testes.

At E14.5, a portion of the *Dnd1^ter/ter^* germ cells showed decreased H3K27me3 levels when compared to wild-type germ cells (Fig. 1A; Fig. S2). Meanwhile, Ezh2 expression was slightly higher in *Dnd1^ter/ter^* germ cells than in the wild-type germ cells at E14.5 and E12.5 (Fig. 2A,B; Fig. S4). One possible reason why *Dnd1^ter/ter^* germ cells with low H3K27me3 signals disappear between E14.5 and E16.5 is that they are undergoing apoptosis during that period. To test this possibility, we investigated apoptosis in the germ cells in E14.5 testes and, indeed, we found that more *Dnd1^ter/ter^* germ cells underwent apoptosis when compared to the wild-type germ cells (Fig. S7A-E). In addition, apoptotic *Dnd1^ter/ter^* germ cells showed lower levels of H3K27me3 than non-apoptotic germ cells (Fig. S7F-H). These results suggested that the *Dnd1^ter/ter^* germ cells with decreased H3K27me3 signals were apoptotic at E14.5 and were subsequently lost.

### Dnd1 interacts with the 3′-UTR of *Ezh2* mRNA and suppresses the inhibitory effect of miR-26a on *Ezh2* mRNA

**Fig. 3.**
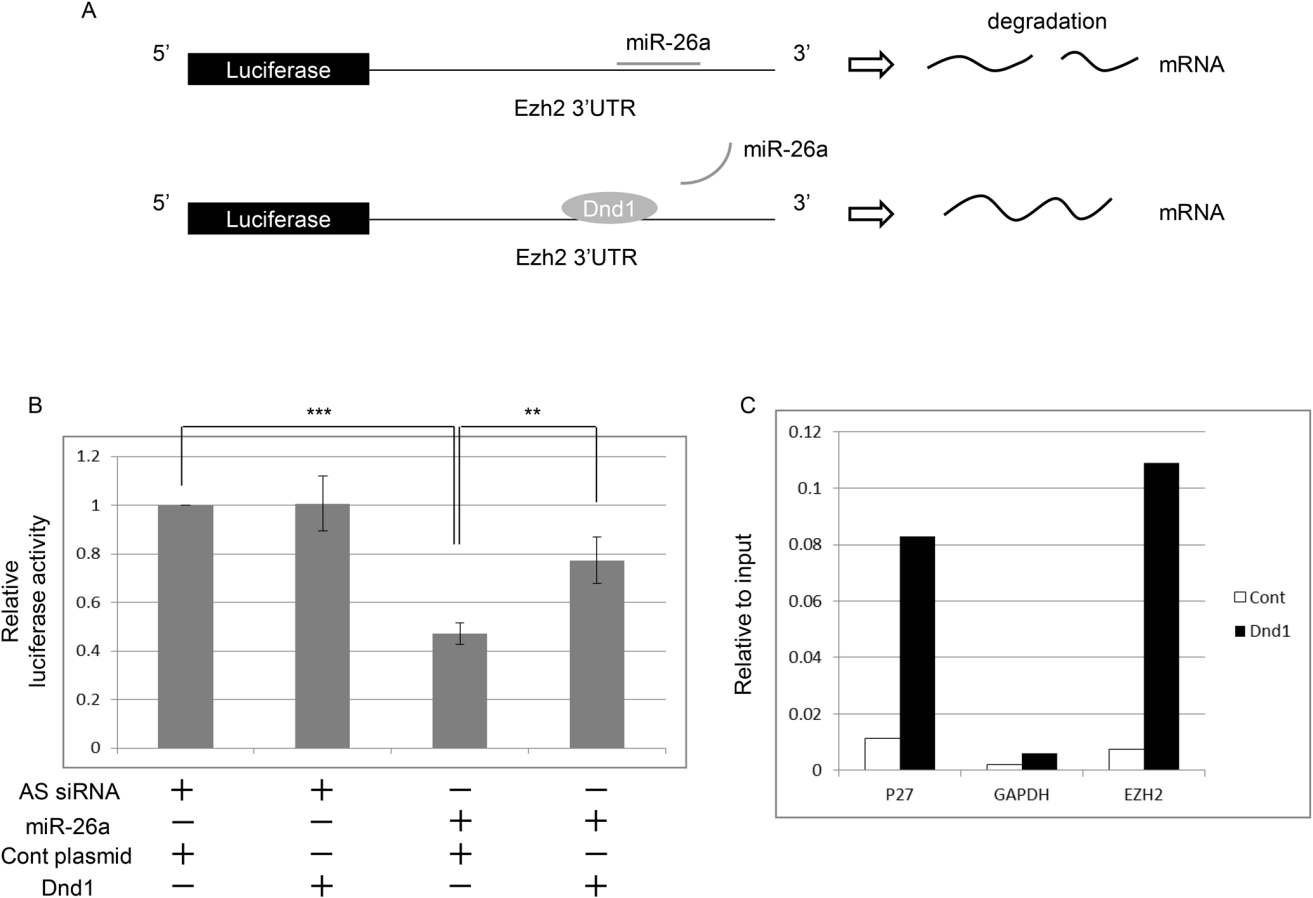
**Dnd1 binds to the 3′-UTR of *Ezh2* mRNA, and suppresses the inhibitory effect of miR-26a on *Ezh2* mRNA.** (A) A schematic representation of the effect of Dnd1 on the luciferase (*luc*) 3′-UTR of an Ezh2 reporter. In the absence of Dnd1, miR-26a associates with the 3′-UTR of *Ezh2* mRNA and suppresses the expression of luciferase; in contrast, in the presence of Dnd1, the binding of miR-26a is inhibited, and luciferase activity is increased. (B) An expression vector containing mouse *Dnd1*, siRNA corresponding to a mature form of mouse miR26a, and the Renilla *luc*-3′-UTR of a mouse Ezh2 reporter vector were co-transfected into HEK293T cells. As a negative control for miR-26a and the Dnd1 expression vector, AllStras (AS) siRNA and an empty vector, respectively, were used. Renilla luciferase activity was normalized to the firefly luciferase activity that was expressed from the same vector, and the normalized luciferase activity of the cells transfected with the AS siRNA and the control empty vector (cont. plasmid) was set as 1. Data were obtained from four independent experiments. **P<0.01, ***P<0.001. (C) RIP assay results show the binding of Dnd1 to *Ezh2* mRNA. An expression vector containing mouse *Dnd1-HA* (Dnd1) or an empty vector (cont) was transfected into HEK293T cells, and the cell extracts were immunoprecipitated using an anti-HA antibody. After purification of the RNA, the amount of precipitated mRNA was quantified by real-time qPCR. As a positive and a negative control, *p27^kip1^* and *Gapdh*, respectively, were used. The vertical axis shows the data relative to the input values. Shown are representative data from two independent experiments. The results of another independent experiment are shown in Fig. S8D.

Dnd1 is an RNA-binding protein that inhibits miRNAs from accessing target mRNAs (Kedde et al., 2007). We observed that the expression of Ezh2 was decreased in the teratoma-forming cells in *Dnd1*-mutant testes when compared to that in wild-type germ cells (Fig. 2D,E). Because a previous study showed that a miRNA, miR-26a, targeted Ezh2 in mouse myogenic C2C12 cells (Wong and Tellam, 2008), and we found that miR-26a was expressed in PGCs (Fig. S8A), we supposed that Dnd1 might interact with the 3′-UTR of *Ezh2* mRNA to inhibit miR-26a-mediated translational repression of Ezh2 in PGCs (Fig. 3A). We examined the 3′-UTR of mouse *Ezh2* mRNA and found a consensus sequence for miR-26a binding (Fig. S9A). We therefore investigated this possibility by using a luciferase assay (Fig. 3A). We transfected siRNA corresponding to mature miR-26a, and a reporter vector of the 3′-UTR of mouse *Ezh2* linked to a luciferase (*luc*) gene into HEK293T cells. As expected, we found that miR-26a significantly inhibited luciferase activity (Fig. 3B). Then, we simultaneously transfected an expression vector of mouse *Dnd1*, miR-26a, and the *luc* reporter vector, and found that Dnd1 rescued the luciferase activity (Fig. 3B).

Next, we investigated whether Dnd1 directly interacted with *Ezh2* mRNA. We performed RNA-binding protein immunoprecipitation (RIP) using HEK293T cells transfected with hemagglutinin (HA)-tagged mouse Dnd1. Although HEK293T is a human cell line, the 3′-UTR of *Ezh2*, as well as *p27* and *Gapdh* mRNA, is highly conserved between mouse and human (Fig. S9B), and the expression of Ezh2 was detected in the HEK293T cells (Fig. S8B). As such, mouse Dnd1 was expected to interact with human *Ezh2* mRNA in HEK293T cells. We found that Dnd1-HA was enriched in the 3′-UTR of *Ezh2* mRNA as well as the *p27^kip1^* mRNA that was used as a positive control, but not in the *Gapdh* mRNA that was used as the negative control (Cook et al., 2011) (Fig. 3C; Fig. S8C,D). These results indicated that Dnd1 directly interacted with the 3′-UTR of *Ezh2* mRNA, and maintained its expression by inhibiting miR-26a.

### *Ccnd1* is targeted by H3K27me3 and is expressed in the teratoma-forming cells in *Dnd1*^ter/ter^ embryonic testes

**Fig. 4.**
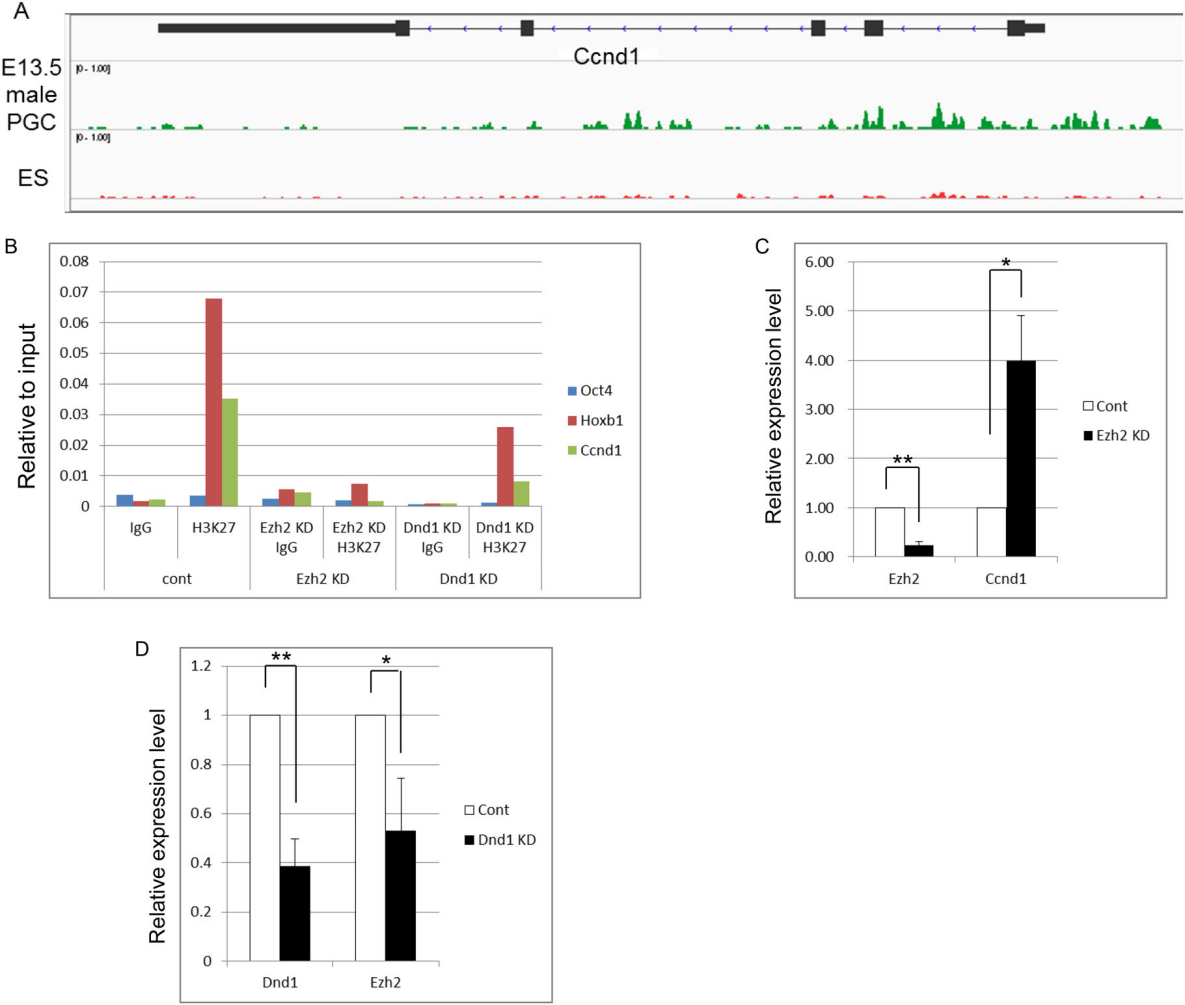
**Ccnd1 is a target of Ezh2.** (A) Enrichment of H3K27me3 at the *Ccnd1* locus in PGCs and ES cells. Representative images of the H3K27me3 ChIP-seq read density at the *Ccnd1* promoter in E13.5 male PGCs (green) and ES cells (red). ChIP-seq data (SRX149169 and SRX186071) were visualized using the Integrative Genomics Viewer (http://software.broadinstitute.org/software/igv/). H3K27me3 was enriched at the transcription start site of *Ccnd1* in E13.5 male PGCs, but not in ES cells. (B) *Ezh2* KD and *Dnd1* KD reduced the enrichment of H3K27me3 at *Ccnd1* in ES cells. The histogram shows the ratios of the immunoprecipitated chromatin to the input chromatin determined by ChIP-qPCR analysis using the anti-H3K27me3 antibody. *Oct4* and *Hoxb1* were shown to be a negative locus and a positive locus, respectively. Shown are representative data from two independent experiments. The results of another independent experiment are shown in Fig. S8E. (C) Upregulation of *Ccnd1* expression by *Ezh2* KD in ES cells. The expression of *Ezh2* and *Ccnd1* was determined by real-time qPCR. (D) Downregulation of *Ezh2* expression by *Dnd1* KD in ES cells. The expression of *Dnd1* and *Ezh2* was determined by real-time qPCR. An empty vector was transfected as a control. Data were obtained from four independent experiments (C,D). *P<0.05, **P<0.01.

**Fig. 5.**
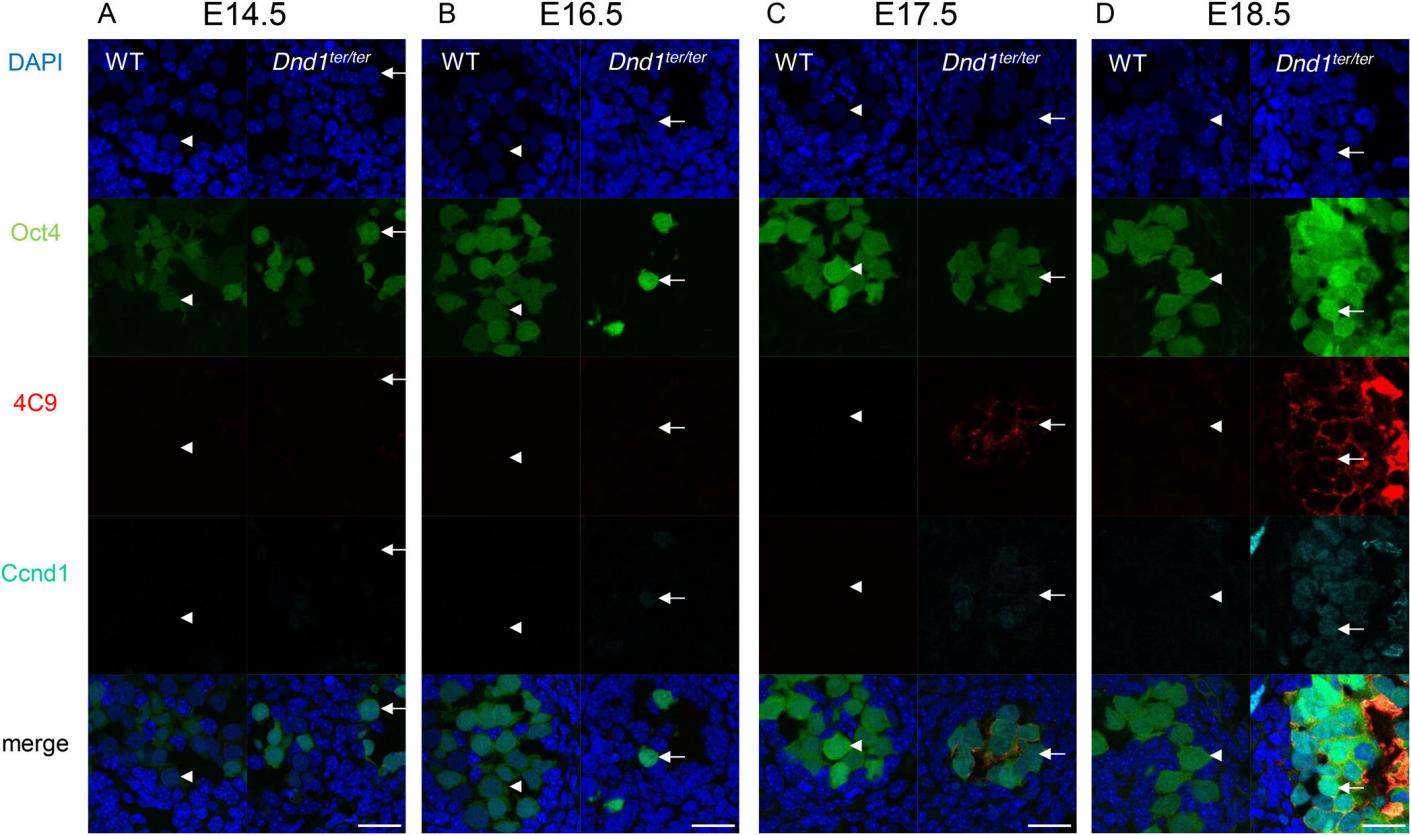
**Ccnd1 expression in the teratoma-forming cells in *Dnd1^ter/ter^* testes, and in germ cells in *Dnd1^ter/ter^* testes and in wild-type/*Dnd1^ter/+^* (WT) testes at E14.5 to E18.5.** (A,B) In Oct4-ΔPE-GFP-positive germ cells in WT (arrowheads) and *Dnd1^ter/ter^* (arrows) testes at E14.5 (A) and E16.5 (B), the expression of Ccnd1 was undetectable. (C,D) 4C9-positive teratoma-forming cells in *Dnd1^ter/ter^* testes (arrows) weakly expressed Ccnd1 at E17.5 (C), then more strongly expressed Ccnd1 at E18.5 (D); in contrast, Ccnd1 was undetectable in GFP-positive germ cells in WT testes (arrowheads) at both E17.5 and E18.5. Scale bars: 25 µm.

We next attempted to identify possible target genes of H3K27me3 that enhance teratoma formation in embryonic germ cells. We re-examined previous chromatin immunoprecipitation sequencing (ChIP-seq) data on H3K27me3 in E13.5 PGCs and embryonic stem (ES) cells (Ng et al., 2013), and selected pluripotency-associated or cell cycle-related gene loci in which H3K27me3 was enriched in PGCs when compared to ES cells. Among them, we chose *Ccnd1* as a promising candidate (Fig. 4A), because Ccnd1 is known to promote testicular teratoma formation (Lanza et al., 2016). Ccnd1 expression is likely repressed via H3K27me3 in embryonic germ cells, and, consistent with this idea, Ccnd1 was upregulated with the decrease in H3K27me3 in 4C9-positive teratoma-forming cells in *Dnd1^ter/ter^* testes between E17.5 and E18.5, and it was not expressed in earlier *Dnd1*-deficient germ cells or in wild-type germ cells (Fig. 5A-D). To gain further information on the roles of Dnd1 and Ezh2 in controlling *Ccnd1* expression, we carried out chromatin immunoprecipitation quantitative polymerase chain reaction (ChIP-qPCR) and reverse transcription quantitative polymerase chain reaction (RT-qPCR) using ES cells after Ezh2 and Dnd1 knockdown (KD). We found that the enrichment of H3K27me3 in the *Ccnd1* locus decreased after Dnd1 KD as well as Ezh2 KD (Fig. 4B; Fig. S8E). In addition, *Ccnd1* was upregulated by Ezh2 KD, while *Ezh2* was downregulated by Dnd1 KD in ES cells (Fig. 4C,D). These results are consistent with the idea that Dnd1 maintains *Ezh2* expression and that Ezh2 represses *Ccnd1* expression via H3K27me3.

### The process of PGC reprogramming into pluripotent stem cells in culture mimics the conversion of embryonic testicular germ cells into teratoma-forming cells

**Fig. 6.**
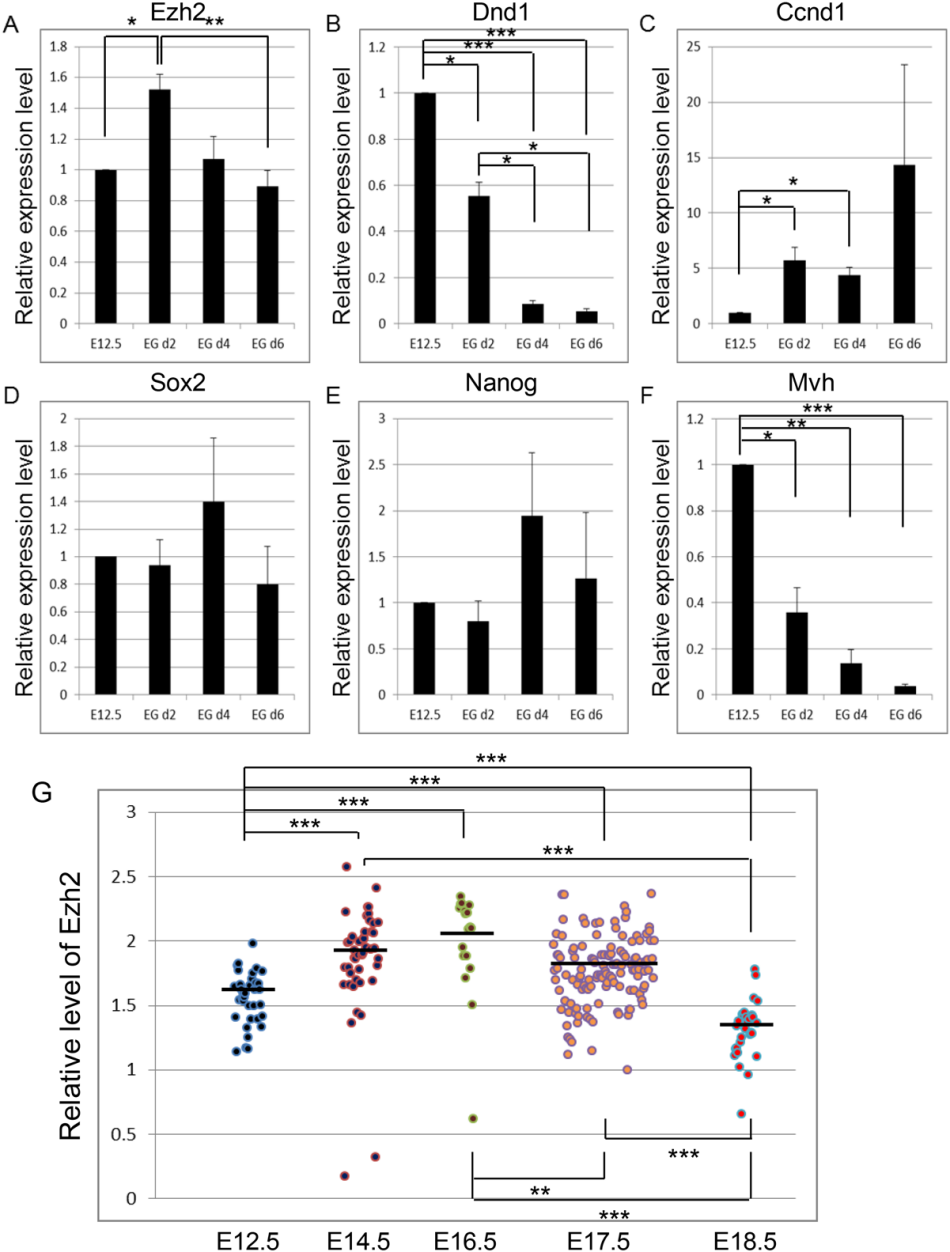
**Expression of teratoma-related genes during PGC reprogramming in culture.** (A-F) Purified E12.5 PGCs (E12.5) of Oct4-ΔPE-GFP transgenic embryos were cultured in the medium for PGC reprogramming without a feeder layer for 2, 4, or 6 days (EG d2, EG d4 or EG d6). The expressions of Ezh2 (A), Dnd1 (B), Ccnd1 (C), Sox2 (D), Nanog (E), and Mvh (F) were determined by RT-qPCR. (G) The expression of Ezh2 in germ cells at E12.5 to E16.5 and in teratoma-forming cells at E17.5 and E18.5 in *Dnd1^ter/ter^* testes. The plots show the relative intensity of the fluorescent signals of Ezh2 in germ cells or teratoma-forming cells in comparison to those in the surrounding somatic cells based on the data shown in Figs 1 and 2. Data were obtained from three (d4 and d6) and four (d2 and d4) independent experiments (A-F). *P<0.05, **P<0.01, ***P<0.001.

We next attempted to elucidate the Dnd1-related molecular pathway that regulates the conversion of embryonic testicular germ cells into teratoma-forming cells. Because teratoma develops from germ cell-derived pluripotent stem cells, we first tested whether changes in the expression of genes during PGC reprogramming into pluripotent stem cells (Matsui et al., 1992; Resnick et al., 1992) mimic those in embryonic germ cells in *Dnd1^ter/ter^* testes. We cultured E12.5 PGCs of wild-type embryos in medium for PGC reprogramming (Matsui et al., 2014). The expression of *Ezh2* was upregulated during the first 2 days in culture, but it subsequently decreased by day 6 (Fig. 6A). In *Dnd1^ter/ter^* testicular germ cells, the relative expression levels of Ezh2 in comparison to those in the adjacent gonadal somatic cells slightly increased between E12.5 and E14.5, and then gradually decreased by E18.5 in the teratoma-forming cells. The downregulation of Mvh (Fig. 6F; Fig. S5), upregulation of Ccnd1 (Fig. 5, Fig. 6C), and maintenance of Sox2 (Fig. 6D; Fig. S1) were also correlated between the cultured PGCs and the teratoma-forming *Dnd1^ter/ter^* testicular germ cells. In addition, the decreased expression of Dnd1 in cultured PGCs in the condition for PGC reprogramming (Fig. 6B) was consistent with its negative influence on the development of teratoma-forming cells. The similar changes in the expression of these molecules suggested that PGC reprogramming partially, at the least, mimics the conversion of *Dnd1^ter/ter^* germ cells into teratoma-forming cells.

### Overexpression of *Ezh2* and KD of *Ccnd1* repress PGC reprogramming

**Fig. 7.**
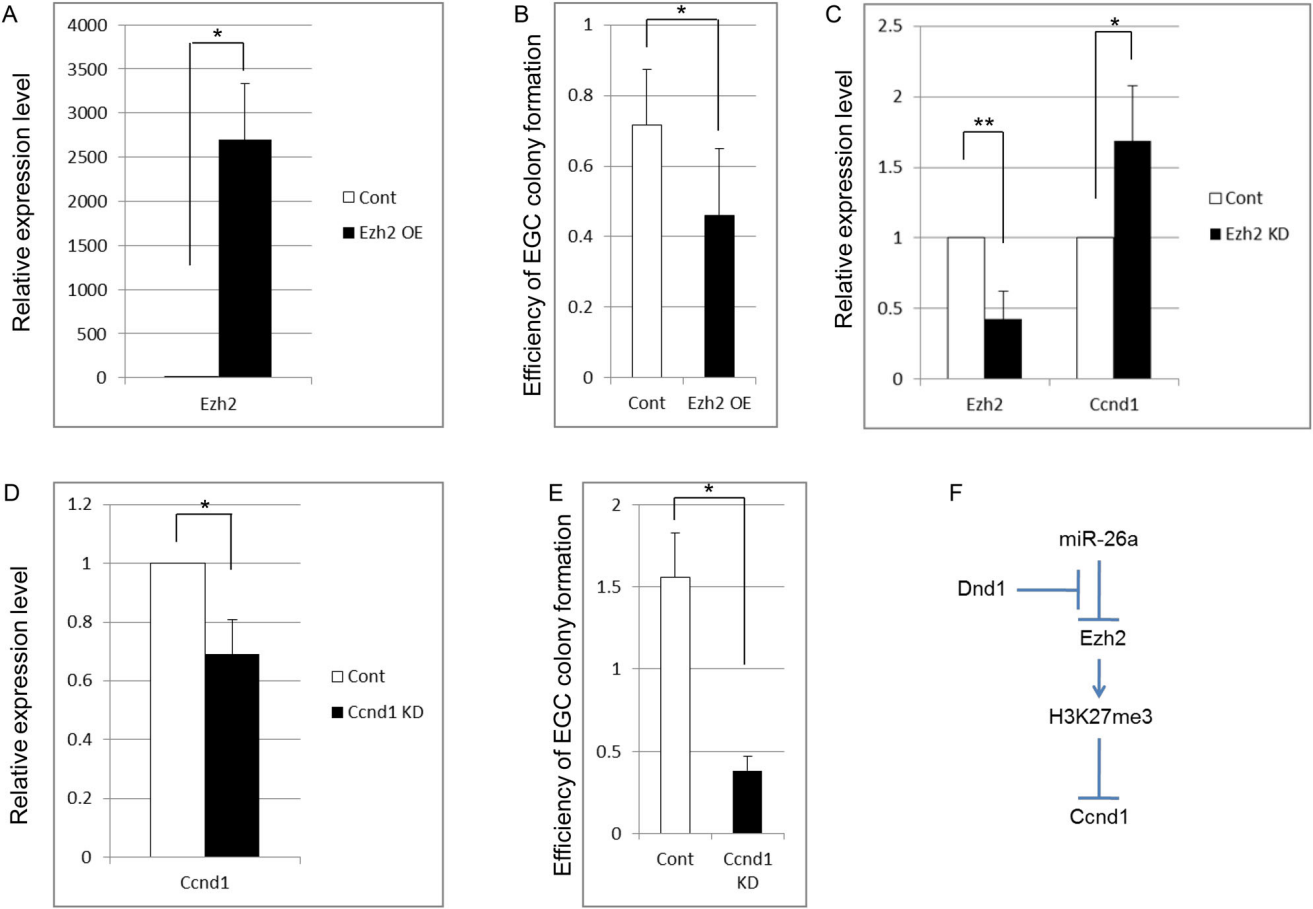
**Ezh2 OE and Ccnd1 KD repress the reprogramming of PGCs in culture.** (A,B) The expression of Ezh2 (A) and efficiency of PGC reprogramming in Ezh2 OE PGCs. (C) The expression of Ezh2 and Ccnd1 in Ezh2 KD PGCs. (D,E) The expression of Ccnd1 (D) and efficiency of PGC reprogramming (E) in Ccnd1 KD PGCs. Purified E12.5 PGCs of Oct4-ΔPE-GFP transgenic embryos were infected with Lentivirus vectors for the OE or KD of Ezh2 or Ccnd1, then cultured in the medium for PGC reprogramming without a feeder layer for 2 days. Expression levels were determined by RT-qPCR. An empty vector was infected as a control. For PGC reprogramming, virus vector-infected purified E12.5 PGCs were cultured with Sl/Sl4-m220 feeder cells. (F) Schematic representation of the linkage between Dnd1 and Ccnd1. Data were obtained from three (B,E) and four (A,C,D) independent experiments. *P<0.05, **P<0.01.

The downregulation of Ezh2 and upregulation of Ccnd1 in the teratoma-forming cells in *Dnd1^ter/ter^* testes in comparison to wild-type germ cells (Figs 2 and 5), and the upregulation of *Ccnd1* and its decreased H3K27me3 due to Ezh2 KD in ES cells (Fig. 4) suggested that the downregulation of Ezh2 due to Dnd1 deficiency leads to the conversion of germ cells into teratoma-forming cells via the upregulation of Ccnd1 expression in *Dnd1^ter/ter^* testes. To gain insight into this possibility, we examined the roles of Ezh2 and Ccnd1 in the reprogramming of PGCs. We found that Ezh2 overexpression (OE) and Ccnd1 KD decreased the efficiency of PGC reprogramming (Fig. 7A,B,D,E). In addition, Ccnd1 expression was increased by Ezh2 KD (Fig. 7C). These results are consistent with those of the ES cells (Fig. 4C). Taken together, our results suggested that Ccnd1 expression, which is controlled by Ezh2, is critical for the conversion of PGCs into pluripotential stem cells in culture. As was mentioned above, PGC reprogramming in culture may mimic the development of teratoma-forming cells in *Dnd1^ter/ter^* testes, and our results support the idea that a similar molecular linkage may be involved in the teratoma-forming cells in embryos.

## DISCUSSION

Our results suggested that the expression of a cell cycle gene, *Ccnd1,* was upregulated by Dnd1 deficiency via the loss of Ezh2 expression and of H3K27me3 in *Dnd1^ter/ter^* testicular germ cells (Fig. 7F), and this molecular pathway may play a role in testicular teratoma development in *Dnd1^ter/ter^* testes. Ccnd1 forms a complex with cyclin-dependent kinase (CDK) 4/6, and enhances the G1-S transition of the cell cycle in response to mitotic signals (Deshpande et al., 2005). A previous study reported that Ccnd1 expression was undetectable in embryonic germ cells, but was aberrantly expressed in E15.5 testicular germ cells in the teratoma-susceptible M19 mouse strain, and *Ccnd1* knock-out M19 mice showed a decreased incidence of teratoma (Lanza et al., 2016). In the present study, we found that Ezh2 OE or Ccnd1 KD in E12.5 PGCs repressed their reprogramming into pluripotent stem cells in culture (Fig. 7), which may mimic the development of teratoma-forming cells (Fig. 6). Taken together, the results suggested that the downregulation of Ccnd1 caused by Dnd1-mediated expression of Ezh2 may be crucial for the suppression of teratoma development in germ cells.

In our analysis, clusters of teratoma-forming cells in which Ccnd1 expression was upregulated were obvious in *Dnd1^ter/ter^* testes at E17.5 (Fig. 5). Previous studies have detected teratoma-forming cell clusters in *Dnd1^ter/ter^* testes as early as E16.5 by analyzing E-cadherin expression (Cook et al., 2011) and Ccnd1 expression in E15.5 testicular germ cells in M15 mice (Lanza et al., 2016). In the present study, we identified teratoma-forming cells as 4C9-positive cells, and 4C9 is likely upregulated at slightly later stages of teratoma development than E-cadherin. Meanwhile, we detected only scattered but not cluster-forming Oct4-ΔPE-GFP-positive cells also expressing Sox2 in *Dnd1^ter/ter^* testis at E16.5 (data not shown). It is also possible that subtle differences in genetic background of our *Dnd1^ter/ter^*/ Oct4-ΔPE-GFP mice from that in previous studies might cause slightly later onset of teratoma development. Regarding the expression of Ccnd1, earlier expression was seen in M19 mice than in our *Dnd1^ter/ter^* mice, but this difference may have been due to strain difference and/or the possibility that the Ccnd1 immunostaining in our experiments was less sensitive. In addition, the teratoma-forming germ cells in *Dnd1*-mutant embryos were morphologically distinguished as early as E14 (Rivers and Hamilton, 1986), suggesting that changes in the molecular markers as well as cellular behaviors, including cell interaction to form cell clusters, may occur after the initial intercellular morphological changes during the development of teratoma-forming cells.

Dnd1-induced downregulation of the expression of CDK inhibitors in embryonic germ cells may also be involved in inhibiting teratoma formation. Although the involvement of *p27^kip1^* and *p21^cip1^*, which encode the CDK inhibitors p27^kip1^ and p21^cip1^, in the regulation of teratoma formation is currently unclear, in embryonic testes, the expression of p27^kip1^ and p21^cip1^ causes embryonic germ cells to enter a period of mitotic arrest at E14.5 (Western et al., 2008). *p27^kip1^* and *p21^cip1^* mRNAs are targets of Dnd1 (Cook et al., 2011; Zhu et al., 2011), and the expression of *p27^kip1^* and *p21^cip1^* mRNAs is abnormally repressed in *Dnd1^ter/ter^* germ cells at E14.5 (Cook et al., 2011). Our results from the present study suggest that *Ccnd1* is controlled by Dnd1 via epigenetic regulation, and it is involved in teratoma formation. Among pluripotent stem cells, a very low proportion of cells are found to be in the G0/G1 phase, whereas many are in the S phase (Singh and Dalton, 2009; Kareta et al., 2015). Thus, it is possible that the teratoma-forming cells may start to alter their cell cycle to resemble that of pluripotent stem cells.

We also showed via a reporter assay in HEK293T cells that Dnd1 maintained the expression of Ezh2 by inhibiting miR26a on the 3′UTR of Ezh2 mRNA (Fig. 3); this result was consistent with a previously reported function of Dnd1 on target mRNAs (Kedde et al., 2007). A recent study also indicated that Dnd1 destabilizes target mRNAs via the recruitment of the CCR-NOT deadenylase complex in PGCs and spermatogonial stem cells (Yamaji et al., 2017), suggesting that Dnd1 either stabilizes or destabilizes different target mRNAs via distinct mechanisms in germ cells.

*Dnd1^ter/ter^* results in a significant decrease in PGC numbers as early as E8.5 (Sakurai et al., 1995). Consistent with this observation, we found that the number of germ cells was much lower in *Dnd1^ter/ter^* embryonic gonads than in wild-type gonads at E12.5, and at E16.5, the number was even lower (Fig. S6). In addition, a high proportion of *Dnd1^ter/ter^* germ cells underwent apoptosis (Fig. S7E), indicating that the decrease in *Dnd1^ter/ter^* germ cell numbers was partly due to apoptosis. These results suggested that a portion of the surviving germ cells transform into teratoma-forming cells in *Dnd1^ter/ter^* testes by E17.5.

The identification of additional genes other than cell cycle-related genes under the control of Dnd1 would be of interest in future experiments. In addition, the possible involvement of epigenetic control, including the repression of cell cycle genes by H3K27me3, in teratoma development in human is also an important subject for further studies.

## MATERIALS AND METHODS

### Mice and genotyping

Oct4-ΔPE-GFP transgenic mice with the B6D2F1 background (Yoshimizu et al., 1999) were backcrossed to 129/Sv/*Dnd1*^ter/+^ mice obtained from The Jackson Laboratory for more than 10 generations to establish a congenic strain of 129Sv/*Dnd1*^ter/+^/Oct4dΔPE-GFP mice. Female and male 129Sv/*Dnd1*^ter/+^/Oct4-ΔPE-GFP mice were mated in the afternoon, and the presence of vaginal plugs was checked the next morning. For PGC culture experiments, MCH females (Japan SCL) were mated with Oct4dΔPE-GFP males. The day on which a plug was found was considered to be E0.5. DNA was extracted from the tail of the embryos, and was genotyped using the primer set TerF:5′-TCCAGGAGACACTGCTGCTA-3′ and TerR:5′- TTCAGGAACTCCACTTGTGC-3′ according to the protocol provided on The Jackson Laboratory website (https://www2.jax.org/protocolsdb/f?p=116:5:0::NO:5:P5_MASTER_PROTOCOL_ID,P5_JRS_CODE:2172,000091). The mice were kept and bred in the Animal Unit of the Institute of Development, Aging and Cancer, Tohoku University, which is a controlled-environment and pathogen-free facility, according to the guidelines for the care and use of experimental animals defined by the facility. Animal protocols were reviewed and approved by the Tohoku University Animal Studies Committee.

### Immunohistochemistry

Embryonic gonads were collected from embryos on E12.5 to E18.5, and were fixed for 3 h in 2% paraformaldehyde at 4°C. Fixed gonads were then soaked in 10% sucrose for 1 h at 4°C, and then in 20% sucrose overnight at 4°C. Samples were embedded in OCT compound (Sakura Finetek, Torrance, USA), and were sectioned at 8 μm. The sections were blocked in 5% skim milk/1% Triton X-100 in phosphate-buffered saline, and were incubated overnight at 4°C in the primary antibodies diluted in 1% skim milk/0.1% Triton X-100 in phosphate-buffered saline. Antibodies included rat anti-4C9 (1:100 dilution; Yoshinaga et al., 1991), rat anti-TRA98 (1:500 dilution; Tanaka et al., 1997), rabbit anti-mouse Vasa (1:500 dilution; Toyooka et al., 2000), rabbit anti-H3K9me2 (1:500 dilution; 07-441, Millipore), rabbit anti-H3K27me3 (1:500 dilution; 07-449, Millipore), rabbit anti-Ezh2 (1:500 dilution; D2C9, Cell Signaling Technology), rabbit anti-Suz12 (1:500 dilution; D39F6, Cell Signaling Technology), rabbit anti-Ccnd1 (1:500 dilution; ab16663, Abcam), and rabbit anti-Sox2 (1:300 dilution; ab97959, Abcam). After washing, sections were incubated with goat anti-rat Alexa Fluor 568 (1:500 dilution; A11077, Invitrogen) or goat anti-rabbit Alexa Fluor 647 (1:500 dilution; A21244, Invitrogen) antibody for 2 h at 4°C. TdT-mediated dUTP nick-end labeling (TUNEL) staining was performed using the In Situ Cell Death Detection Kit, TMR Red (Roche), according to the manufacturer's instructions. Nuclei were counterstained with 4′,6-diamidino-2-phenylindole. Images were taken using an SP8 Confocal Microscope (Leica Microsystems, Wetzlar, Germany).

### Quantification of fluorescent intensity in the immunostained sections

Fluorescent signals were quantified using an LAS AF Lite program (Leica). The average pixel value in the nucleus of each cell was estimated, and then plotted in graphs. The fluorescent signal intensities of 10 randomly selected somatic cells, 10-30 germ cells in wild-type/*Dnd1^ter/+^* testis, and 10-40 germ cells or teratoma-forming cells in *Dnd1^ter/ter^* testes in each section were measured using a confocal microscope. Sections from three embryos of each genotype were observed; from each embryo of wild-type/*Dnd1^ter/+^* or *Dnd1^ter/ter^* testes, one to two sections or one to three sections, respectively, were obtained. The average value of the somatic cells in each section was set as 1, and the fluorescence values of the germ cells or the teratoma-forming cells were normalized to the values of the somatic cells in the same section.

### Cell culture and luciferase assay

The 3′-UTR of *Ezh2* was PCR-amplified from mouse tail genomic DNA using the primer set F:5′-TGAAGTATGTGGGCATCGAA and R:5′-GCAAGCTGGAAAAACAAAAGC, and was subcloned into a pGEM-T Easy Vector (Promega, Fitchburg, USA). The 3′-UTR of *Ezh2* was then cut off by *Not*I, and was sub-cloned downstream of the Renilla luciferase gene in a psiCHECK2 vector (Promega). An open reading frame of *Dnd1* was amplified from mouse testis cDNAs using the primer set F:5′-TTGAATTCACCATGCAGTCCAAACGGGAGTG and R:5′-TTGAATTCTTAAGCATAATCTGGAACATCATATGGATACTGCTTAACCATAGTACCT. The R (reverse) primer contained the sequence for an HA tag. The PCR product was then subcloned into the *Eco*RI*/Xbal*I site of a pCAGGS vector (Niwa et al., 1991). miR-26a-5p, 5′-UUCAAGUAAUCCAGGAUAGGCU, was an artificial composition obtained from Genosys siRNA Service (Sigma-Aldrich). HEK293T cells were cultured in high glucose Dulbecco's Modified Eagle Medium (DMEM; Gibco) with 10% fetal bovine serum (FBS) in an atmosphere of 5% CO_2_ at 37°C. Then, 500 ng each of the psiCHECK2-Ezh2 3′-UTR vector and of the pCAGGS-Dnd1-HA or an empty vector were co-transfected into HEK293T cells using Lipofectamine 2000 (Invitrogen) with 8 nM miR-26a-5p or AllStars siRNA (Qiagen) as a negative control in a 24-well dish. Cells were collected for the luciferase assay after 48 h of incubation, and a Lumat LB 9507 (Berthold, Bad Wildbad, Germany) was used for the measurements according to the manufacturer's instructions. Data were obtained from four independent experiments.

### RIP assay

pCAGGS-Dnd1-HA or an empty vector was transfected into HEK293T cells. The cells were collected after 48 h in culture, and were used for RNA immunoprecipitation using an RIP Assay Kit (MBL, Nagoya, Japan). Protein A Sepharose CL-4B (GE Healthcare) and mouse anti-HA antibody (12CA5, Roche) were used for immunoprecipitation. RNA was purified and reverse transcribed using SuperScript III Reverse Transcriptase (Invitrogen) with random primers (Promega). For real-time qPCR, the reaction mix (total volume of 20 µl) contained 1 µl of cDNA template, 10 μl of 2× Power SYBR Green PCR Master Mix (Applied Biosystems, Warrington, UK), 8 µl of Milli-Q water, and 1 µl of 20 µM gene-specific forward and reverse primers (*Ezh2*, F:5′-CCCCTCCTCTGAAACAGCTG, R:5′-GCCCACAGTACTCGAGGTTC; *P27^kip1^*, F:5′-GCCCTCCCCAGTCTCTCTTA, R:5′-CTCCCAAGCACCTCGGATTT; *Gapdh*, F:5′-CACCATCTTCCAGGAGCGAC, R:5′-GACTCCACGACGTACTCAGC). qPCR was performed using a CFX Connect Real-Time System (Bio-Rad). The cycling conditions were as follows: 50°C for 2 min (one cycle); 95°C for 2 min (one cycle); and 95°C for 15 s and 60°C for 30 s (45 cycles). For western blotting, mouse anti-HA antibody (1:200 dilution; SC-7293, Santa Cruz Biotechnology) or rabbit anti-Ezh2 antibody (1:1000 dilution; D2C9, Cell Signaling Technology) was used as the primary antibody, and horseradish peroxidase-conjugated anti-mouse IgG or anti-rabbit IgG was used as the secondary antibody at a dilution of 1:5000. Signals were detected by electrochemiluminescence (ELC) (Bio-Rad), and images were taken using a LAS-3000 (Fujifilm, Tokyo, Japan).

### PGC and ES cell cultures, Ezh2 OE, Ezh2 KD, Dnd1 KD and Ccnd1 KD

To construct an Ezh2 OE Lentivirus vector (CSII-EF-Ezh2), the coding region of Ezh2 was PCR amplified from mouse E12.5 PGC cDNA using the F:5′-CCGGTCTCGAGAATTGCCACCATGGGCCAGACTGGG and R:5′-AGAGGATCCGCGGCCTCAAGGGATTTCCATTTCTCG primer set, then subcloned into the CSII-EF-MCS Lentivirus vector. CSII-EF-Ezh2 (6.8 µg), pCAG-HIVgp (4 µg), and pCMV-VSV-G-RSV-Rev (4 µg) were co-transfected into HEK293 T cells using Lipofectamine 3000 (Invitrogen). Ezh2-, Dnd1-, and Ccnd1 KD shRNA vectors (pKLO.1-shEzh2, pKLO.1-shDnd1, and pKLO.1-shCcnd1) were constructed by annealing the pairs of oligo-nucleotides F:5′-CCGGGCACAAGTCATCCCGTTAAAGCTCGAAGCTTTAACGGGATGACTTGTGCTTTTTG and R:5′-AATTCAAAAAGCACAAGTCATCCCGTTAAAGCTCGAGCTTTAACGGGATGACTTGTGC for Ezh2; F:5′-CCGGGTCAGGGTCCGAGGTGTATATCTCGAGATATACACCTCGGACCCTGACTTTTTG and R:5′-AATTCAAAAAGTCAGGGTCCGAGGTGTATATCTCGAGATATACACCTCGGACCCTGAC for Dnd1; and F:5′-CCGGGCATCTACACTGACAACTCTACTCGAGTAGAGTTGTCAGTGTAGATGCTTTTTG and R:5′-AATTCAAAAAGCATCTACACTGACAACTCTACTCGAGTAGAGTTGTCAGTGTAGATGC for Ccnd1, and by subcloning into an *Age*I/*Eco*RI site in a pKLO.1 Lentivirus vector. pKLO.1-shEzh2, pKLO.1-shDnd1, pKLO.1-shCcnd1, or an empty vector (6.8 µg) was co-transfected with pCAG-HIVgp (4 µg) and pCMV-VSV-G-RSV-Rev (4 µg) into HEK293T cells by the calcium phosphate method . The culture medium was changed after 16 h, and then the medium was collected after 48 h. Polyethylene glycol 6000 was added to the collected medium. After incubation overnight at 4°C, virus particles were collected by centrifuging the medium at 2330×***g*** for 30 min at 4°C, and they were re-suspended in 0.5 ml GS medium (Stem Pro 34 SFM, Gibco) containing 2.5% Stem Pro 34 Nutrient, 100 μg/ml transferrin (Sigma-Aldrich), 2 mM L-glutamine (Gibco), 1% penicillin/streptomycin (Sigma-Aldrich), 10% Knock-out Serum Replacement (Gibco), 25 µg/ml insulin (Sigma-Aldrich), 50 µM 2-mercaptoethanol, 20 ng/ml epidermal growth factor (Sigma-Aldrich**)**, 25 ng/ml basic fibroblast growth factor (Sigma-Aldrich), and 1×10^3^/ml leukemia inhibitory factor (Millipore) (Matsui et al., 2014). E12.5 PGCs were purified from Oct4-ΔPE-GFP transgenic male embryos by flow cytometry (S3e Cell Sorter, Bio-Rad); they were subsequently infected with the virus vectors, and cultured in GS medium without a feeder layer in an atmosphere of 5% CO_2_ at 37°C. An empty pKLO.1 vector was used as a control. After 48 h, PGCs were collected, and the RNA was extracted. For KD studies in ES cells, E14tg2a cells were cultured in medium containing G-MEM (Gibco), 10% FBS, 100 µM MEM Non-Essential Amino Acids (Gibco), 1 mM sodium pyruvate (Gibco), 100 µM 2-mercaptoethanol, and 1000 U/ml leukemia inhibitory factor (Millipore). For KD, pKLO.1-shEzh2, pKLO.1-shDnd1, or an empty vector was transfected by lipofection. The transfected cells were cultured for 72 h before harvesting for ChIP-qPCR or RT-qPCR. Using random primers and SuperScript III Reverse Transcriptase (Invitrogen), cDNA was synthesized. 2× Power SYBR Green PCR Master Mix (Applied Biosystems) was used for real-time qPCR in 20 µl of reaction solution containing 1 µl of cDNA, 8 µl of Milli-Q water, and 1 μl of 20 µM gene-specific forward and reverse primers (Ezh2, F:5′-CCCCTCCTCTGAAACAGCTG, R:5′-GCCCACAGTACTCGAGGTTC; Dnd1, F:5′-GCTGCTCAAGTTCAGTACGCAC, R:5′-GAAGTGCTGCTTTAGGTCTGGC; Ccnd1, F:5′-AGTGCGTGCAGAAGGAGATT, R:5′-AGGAAGCGGTCCAGGTAGTT); Sox2, F:5′-GCGGAGTGGAAACTTTTGTCC, R:5′-CGGGAAGCGTGTACTTATCCTT; Nanog, F:5′-GAACGCCTCATCAATGCCTGCA, R:5′-GAATCAGGGCTGCCTTGAAGAG; Mvh, F:5′-GGACGAGATTTGATGGCTTGTGC, R:5′-AGCGACTGGCAGTTATTCCATCC; and Arbp, F:5′-AGATTCGGGATATGCTGTTGGC, R:5′-TCGGGTCCTAGACCAGTGTTC). Arbp was used as an internal control. qPCR was performed using a CFX Connect Real-Time System (Bio-Rad). The cycling conditions were as follows: 50°C for 2 min (one cycle); 95°C for 10 min (one cycle); and 95°C for 15 s and 60°C for 2 min (45 cycles). For PGC reprogramming, the virus-infected PGCs were plated onto Sl/Sl4-m220 feeder cells (Matsui et al., 1991) with GS medium and cultured for 7 days. Pluripotential stem cell colonies were identified by staining for alkaline phosphatase, as previously described (Matsui et al., 1991). The efficiency of PGC reprogramming was determined as a ratio of the number of colonies to every 100 sorted PGCs that were seeded into a culture well.

### Detection of miR-26a in PGCs

The total RNAs of adult mouse kidney and E18.5 PGCs were extracted using an RNeasy Micro Kit (Qiagen). The Mir-X miRNA First-Strand Synthesis and SYBR qRT-PCR kits (Takara Bio USA, Mountain View, USA) were used for reverse transcription and real-time PCR. qPCR was performed using a CFX Connect Real-Time System (Bio-Rad). The cycling conditions were as follows: 95°C for 10 s (one cycle); 95°C for 5 s (one cycle); and 60°C for 20 s (40 cycles). The forward primer for miR-26a was 5′-TTCAAGTAATCCAGGATAGGCT. The mRQ 3′ Primer from the Mir-X kit was used as the reverse primer. A 100-bp PCR product corresponding to miR-26a was confirmed using 2% agarose gel electrophoresis.

### ChIP-qPCR

To crosslink the ES cells, 100,000 ES cells were incubated in 1% formaldehyde at room temperature with gentle inversion for 10 min. The chromatin was fragmented by sonication in a Bioruptor (Diagenode, Liege, Belgium) 30 s ON and 30 s OFF; total processing time of 12 min; output level, medium. Anti-rabbit H3K27me3 antibody (07-449) was obtained from Millipore, and rabbit IgG (2729) was obtained from Cell Signaling Technology. For each immunoprecipitation, 10 µl of Dynabeads Protein A (Invitrogen) was incubated with 1 µg of the indicated antibody in 500 µl of RIPA (50 mM Tris-HCl at pH 8.0, 150 mM NaCl, 1 M ethylenediaminetetraacetic acid, 0.1% sodium dodecyl sulfate, 1% Triton X-100, and 0.1% sodium deoxycholate) overnight at 4°C with rotation, then washed twice with ice-cold RIPA. Half of the fragmented chromatin was incubated with antibody-bound Dynabeads overnight at 4°C with rotation. The enrichment of specific regions in the immunoprecipitated DNAs was analyzed by quantitative PCR with the Power SYBR Green PCR Master Mix (Applied Biosystems). PCR signals were detected using CFX Connect (Bio-Rad). Data were obtained from two independent experiments. *Oct4* and *Hoxb1* were tested as a representative negative and positive control locus, respectively. The sequences of the PCR primers were: Ccnd1, F:5′-GCCAATATTATGCGCCATCT, R:5′-CCACCCCAAATATTCCTCAC; Oct4, F:5′-F:CAAGGCTAGAGGGTGGGATT, R:5′-GTGGAAAGACGGCTCACCTA; and Hoxb1, F:5′-TTTAGAGTACCCACTTTGTAACC, R:5′-GGCTGCTGGACAGGATAC.

### Statistical analysis

Statistical analysis was performed using one-way ANOVA and the Student's *t*-test. *P*<0.05 was considered statistically significant.

## Supplementary Material

Supplementary information
