# Supplementary material for: Dnd1-mediated epigenetic control of teratoma formation in mouse
Source: Biol Open. 2018 Jan 15;7(1):bio032318. doi: 10.1242/bio.032318 (PMC5829515; doi:10.1242/bio.032318)
Supplement: Supplementary information [file biolopen-7-032318-s1.pdf]

# Supplementary figures

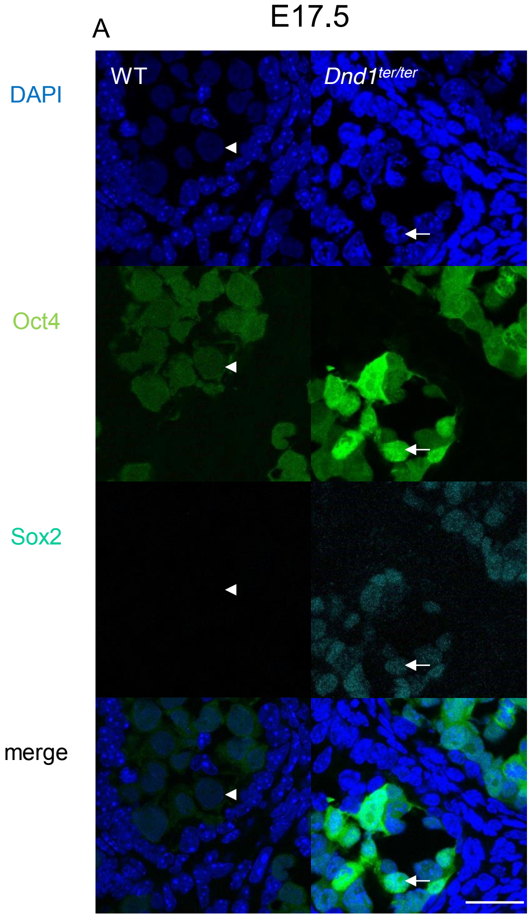


Fig. S1

The expression of Sox2 in Oct4-ΔPE-GFP-expressing teratoma-forming cell clusters (arrows) in *Dnd1*$^{ter/ter}$ testes. Sox2 was undetectable in WT germ cells (arrowheads) at this stage. Scale bar: 25 ⌠m.

Biology Open • Supplementary information

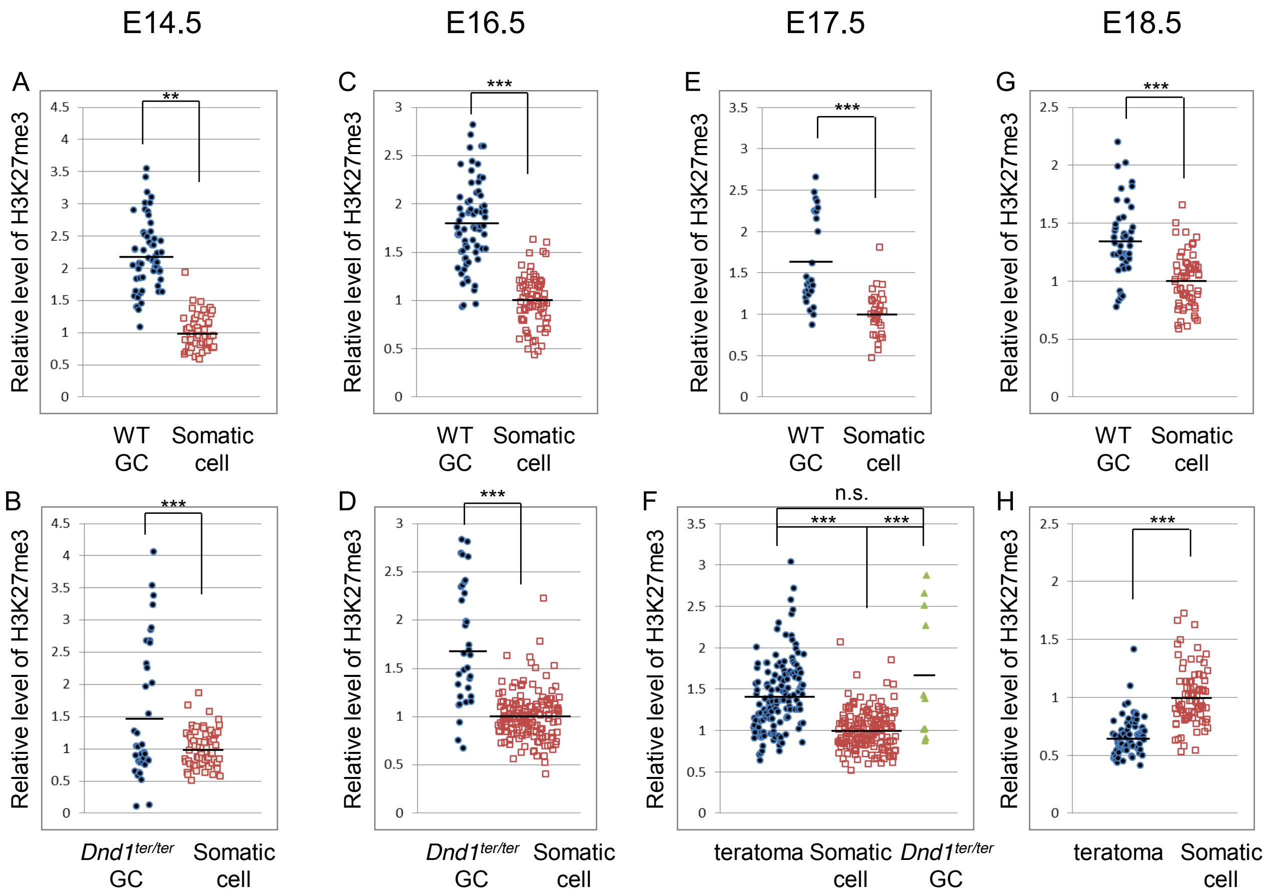


Fig. S2

Quantitative analysis of the H3K27me3 signal intensity in teratoma-forming cells and germ cells relative to the surrounding somatic cells in wild-type or *Dnd1*$^{ter/+}$ (WT) and *Dnd1*$^{ter/ter}$ testes at E14.5 to E18.5. *** P < 0.001; n.s.: not significantly different.

Biology Open • Supplementary information

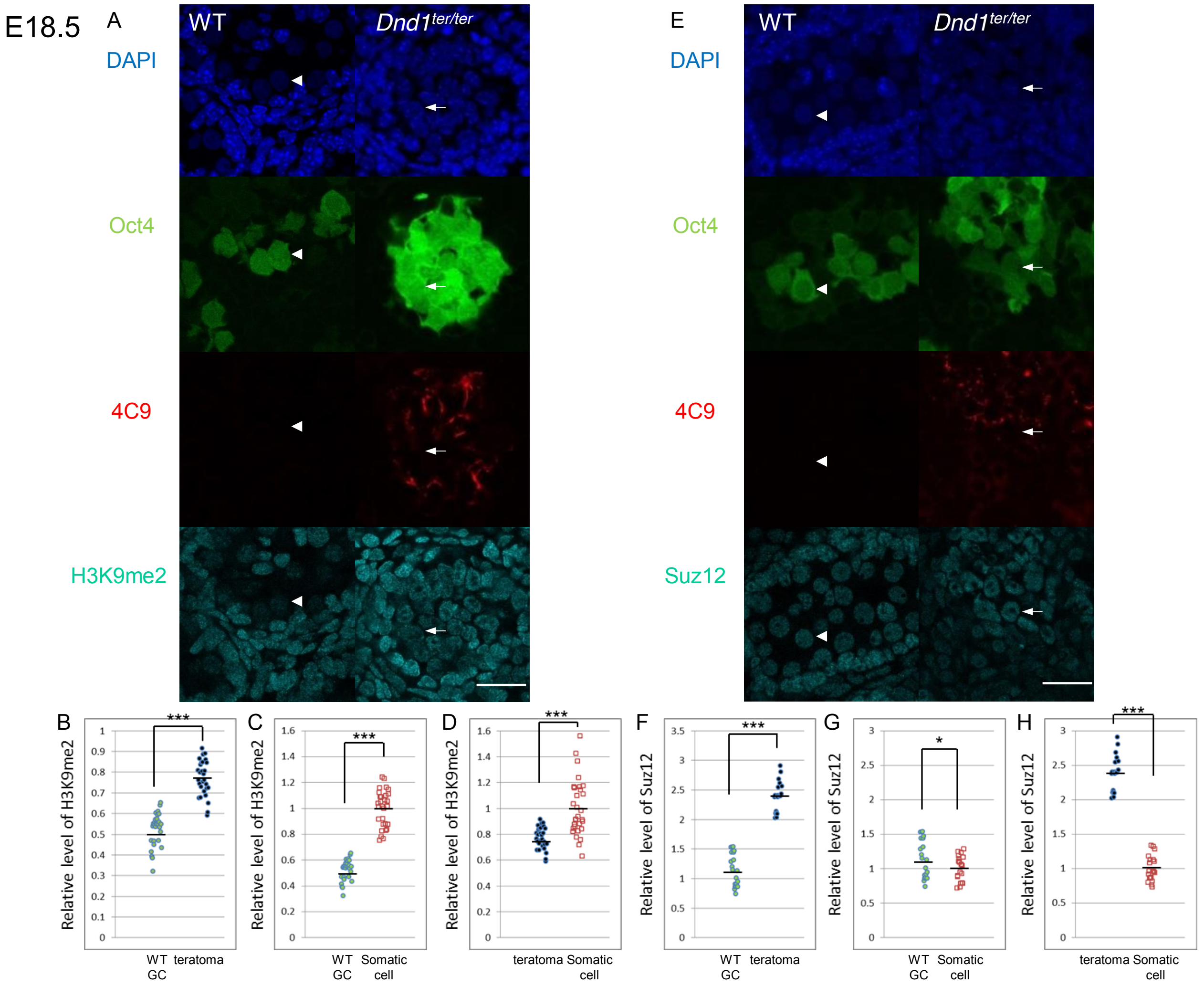


Fig. S3

H3K9me2 and Suz12 expression in teratoma-forming cells and in germ cells in *Dnd1*$^{ter/ter}$ testes and in wild-type or *Dnd1*$^{ter/+}$ testes (WT) at E18.5.

(A, E) Oct4-ΔPE-GFP-positive germ cells (arrowheads) in WT testes showed lower H3K9me2 (A) and Suz12 (E) signal intensities than 4C9-positive teratoma-forming cells (arrows) in *Dnd1*$^{ter/ter}$ testes at E18.5. (B-D, F-H) Quantitative analysis of the H3K9me2 (B-D) and Suz12 (F-H) signal intensities in teratoma-forming cells (teratoma), germ cells (GC), and somatic cells in WT or *Dnd1*$^{ter/ter}$ testes at E18.5. The average signal intensity of 10 randomly selected somatic cells in each section was set as 1, and the signal intensity of each germ cell or teratoma-forming cell relative to the average value of the somatic cells in the same section was estimated (C, D, G, H). B and F show comparisons between the germ cells in WT testes and the teratoma-forming cells in *Dnd1*$^{ter/ter}$ testes. In total, three to five sections from three embryos of each genotype were observed. * $P < 0.05$, *** $P < 0.001$; scale bar: 25 µm.

Biology Open • Supplementary information

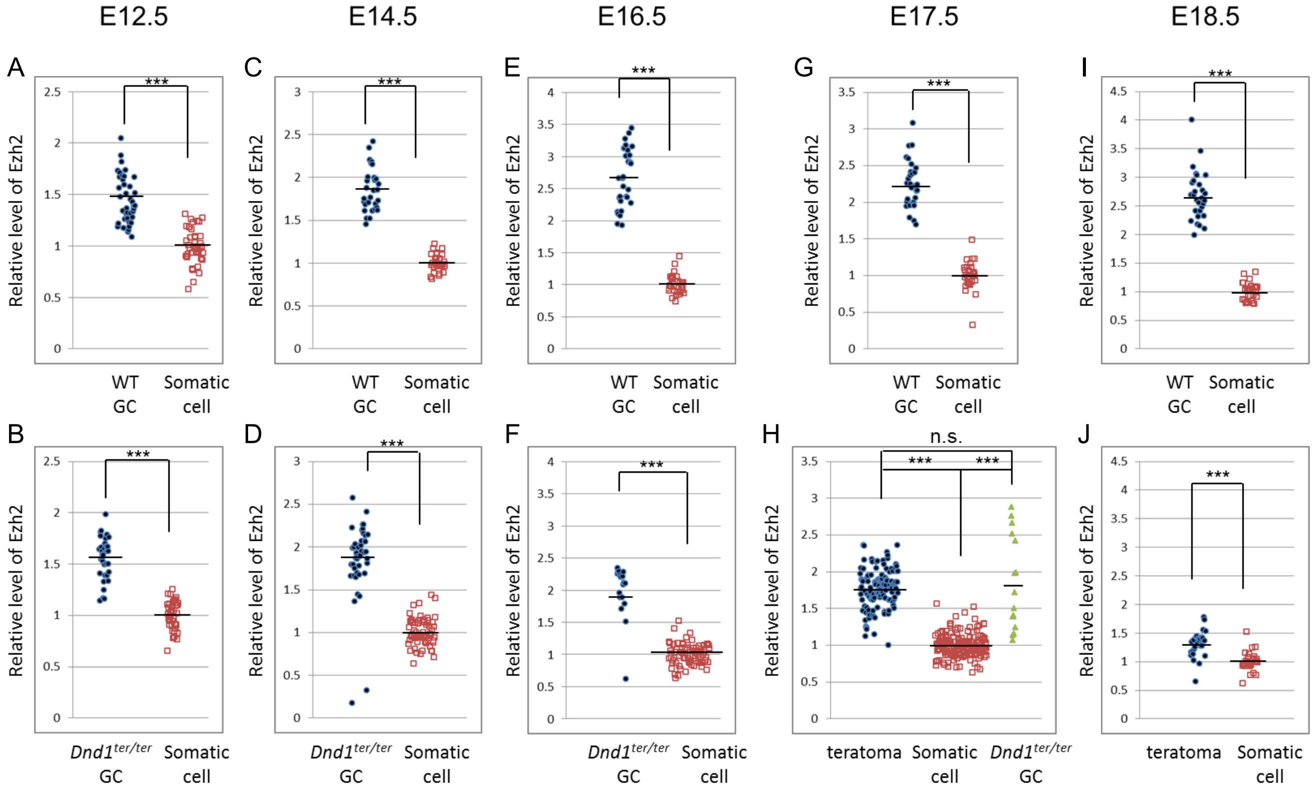


Fig. S4

Quantitative analysis of the Ezh2 signal intensity in teratoma-forming cells (teratoma) and germ cells (GC) relative to the surrounding somatic cells in wild-type or *Dnd1*$^{ter/+}$ (WT) and *Dnd1*$^{ter/ter}$ testes at E12.5 to E18.5. *** P < 0.001; n.s.: not significantly different.

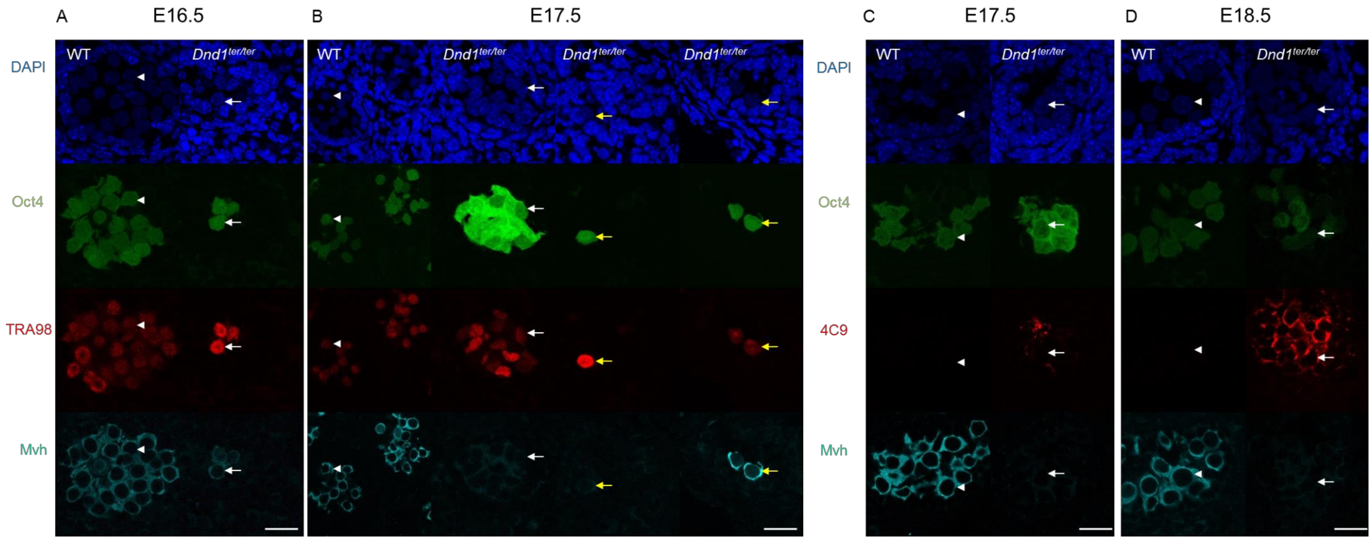


Fig. S5

The expression of Mvh decreased in the teratoma-forming cells in *Dnd1*$^{ter/ter}$ testes.

(A) Oct4-ΔPE-GFP- and TRA98-positive germ cells in WT and *Dnd1*$^{ter/ter}$ testes at E16.5 showed Mvh expression (arrowheads and arrows). (B, C) Oct4-ΔPE-GFP- and TRA98-positive germ cells in WT testes at E17.5 showed Mvh expression (arrowheads in B). GFP- and TRA98-positive (white arrows in B) and GFP- and 4C9-positive (arrows in C) teratoma-forming cells in a cluster in *Dnd1*$^{ter/ter}$ testes at E17.5 showed very low or undetectable levels of Mvh, while a few scattered GFP- and TRA98-positive germ cells expressed Mvh with different intensities (yellow arrows in B) in *Dnd1*$^{ter/ter}$ testes at E17.5. (D) Mvh was undetectable in GFP- and 4C9-positive teratoma-forming cells (arrows) in *Dnd1*$^{ter/ter}$ testes at E18.5. Scale bar: 25 μm.

Biology Open • Supplementary information

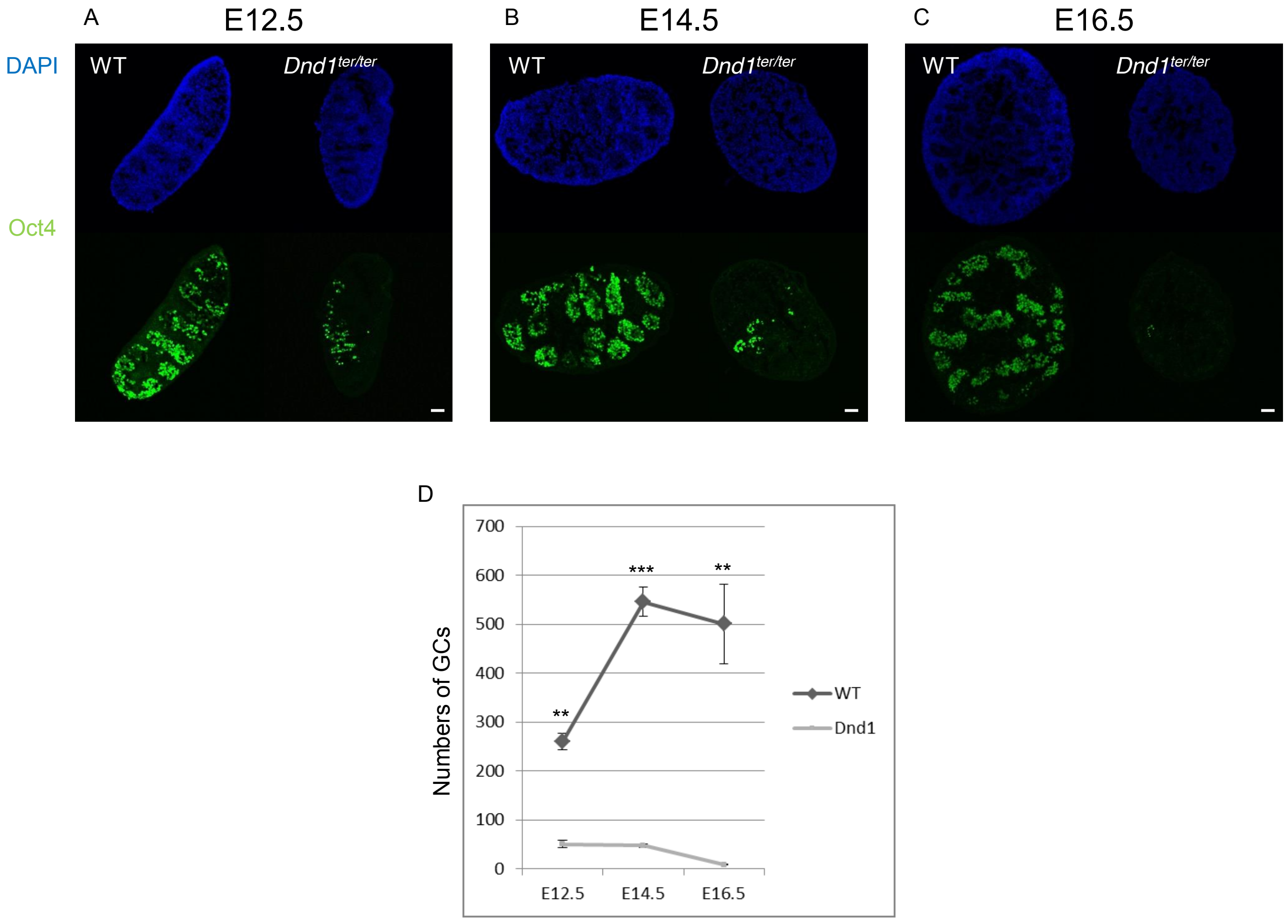


Fig. S6

Change in the number of germ cells in *Dnd1*$^{ter/ter}$ testes during development.

(A) Oct4-ΔPE-GFP-positive germ cells in *Dnd1*$^{ter/ter}$ testes and in wild-type or *Dnd1*$^{ter/+}$ testes (WT) at E12.5, E14.5, and E16.5 are shown. (B) The number of germ cells in each section. In total, three sections from two to three embryos of each genotype were observed. ** P < 0.01, *** P < 0.001; scale bar: 25 μm.

Biology Open • Supplementary information

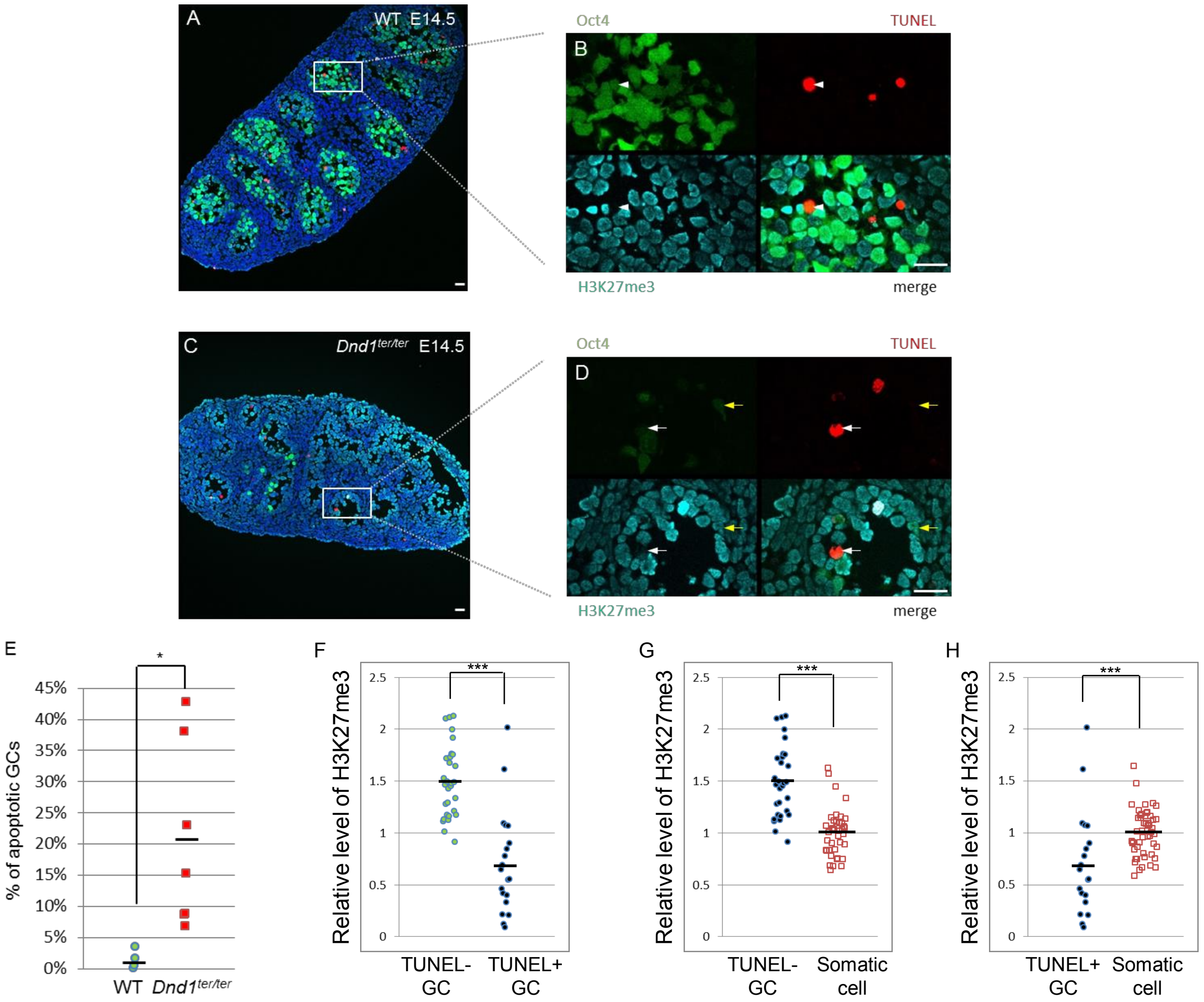


Fig. S7

Germ cells with low levels of H3K27me3 underwent apoptosis in E14.5 *Dnd1*$^{ter/ter}$ testes.

(A, B) Few Oct4-ΔPE-GFP-positive germ cells were TUNEL-positive in wild-type or *Dnd1*$^{ter/+}$ testes (WT) (white arrowheads in B). Panel B shows a higher magnification of the rectangular area shown in panel A. (C, D) TUNEL-positive germ cells in *Dnd1*$^{ter/ter}$ testes exhibited a low H3K27me3 signal intensity (white arrows in D), while TUNEL-negative germ cells exhibited a higher H3K27me3 signal intensity (yellow arrows in D). Panel D shows a higher magnification of the rectangular area shown in panel D. (E) The proportion of TUNEL-positive germ cells was higher in *Dnd1*$^{ter/ter}$ testes than in WT testes. The proportion of TUNEL-positive germ cells among all germ cells in each section is shown on the graph. In total, four sections of WT testes and seven sections of *Dnd1*$^{ter/ter}$ testes from three embryos of each genotype were observed. (F-H) Quantitative analysis of the H3K27me3 signal intensity in TUNEL-positive germ cells (GC) or in TUNEL-negative germ cells relative to the surrounding somatic cells in *Dnd1*$^{ter/ter}$ testes (F). The average signal intensity of 10 randomly selected somatic cells in each section was set as 1, and the signal intensity of each germ cell relative to the average value of the somatic cells in the same section was estimated (G, H). In total, six sections from three embryos were observed. * $P < 0.05$, *** $P < 0.001$; scale bar: 25 μm.

Biology Open • Supplementary information

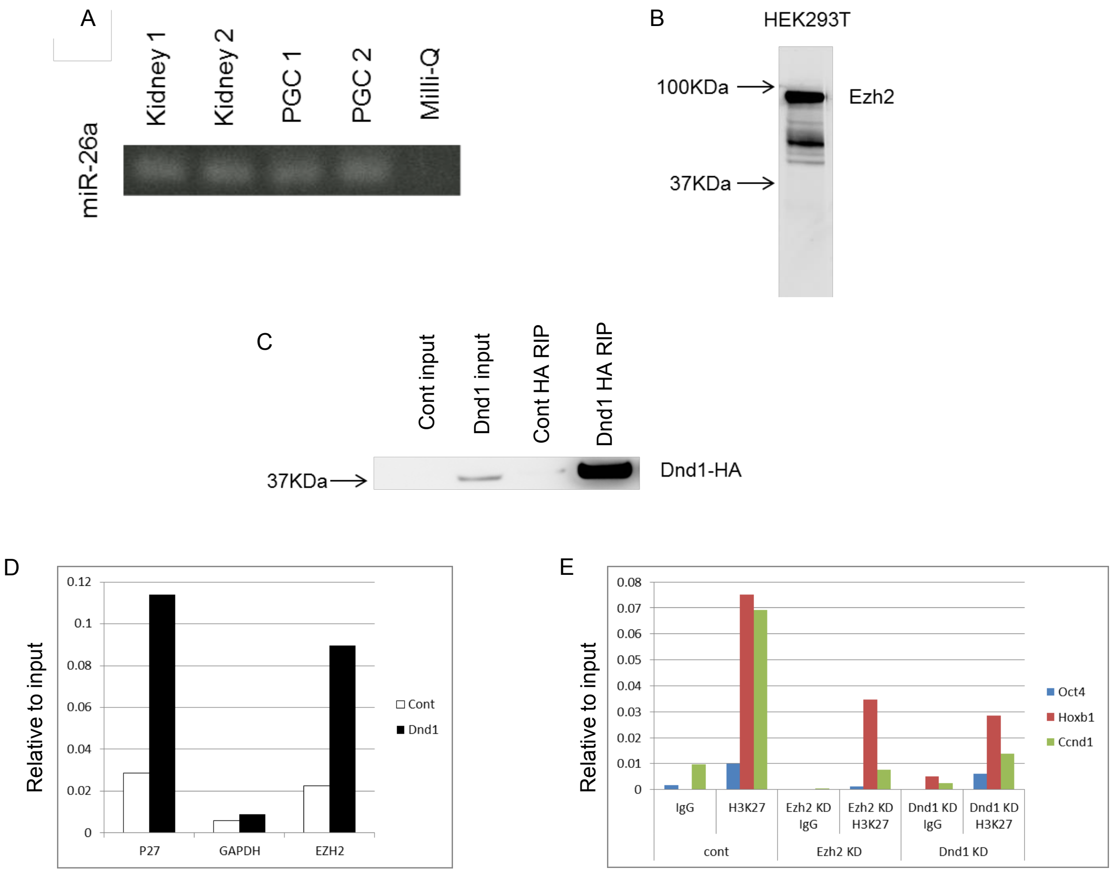


Fig. S8

(A) Detection of miR-26a expression in germ cells at E18.5 by RT-PCR. Adult kidney was used as a positive control (Koga et al., 2015). Data were obtained from two independent samples, and representative data are shown. (B) The expression of Ezh2 in HEK293 cells. Cell extracts were subjected to western blotting with anti-Ezh2 antibody. (C) Enrichment of mouse Dnd1-HA after RIP. The immunoprecipitated cell extract was subjected to western blotting with anti-HA antibody. (D) A replicate of the RIP experiment for the interaction of Dnd1 and Ezh2 mRNA shown in Fig. 3C. (E) A replicate of the ChIP-qPCR for H3K27me3 at the *Ccnd1* locus in Ezh2 KD or Dnd1 KD ES cells shown in Fig. 4B.

Biology Open • Supplementary information

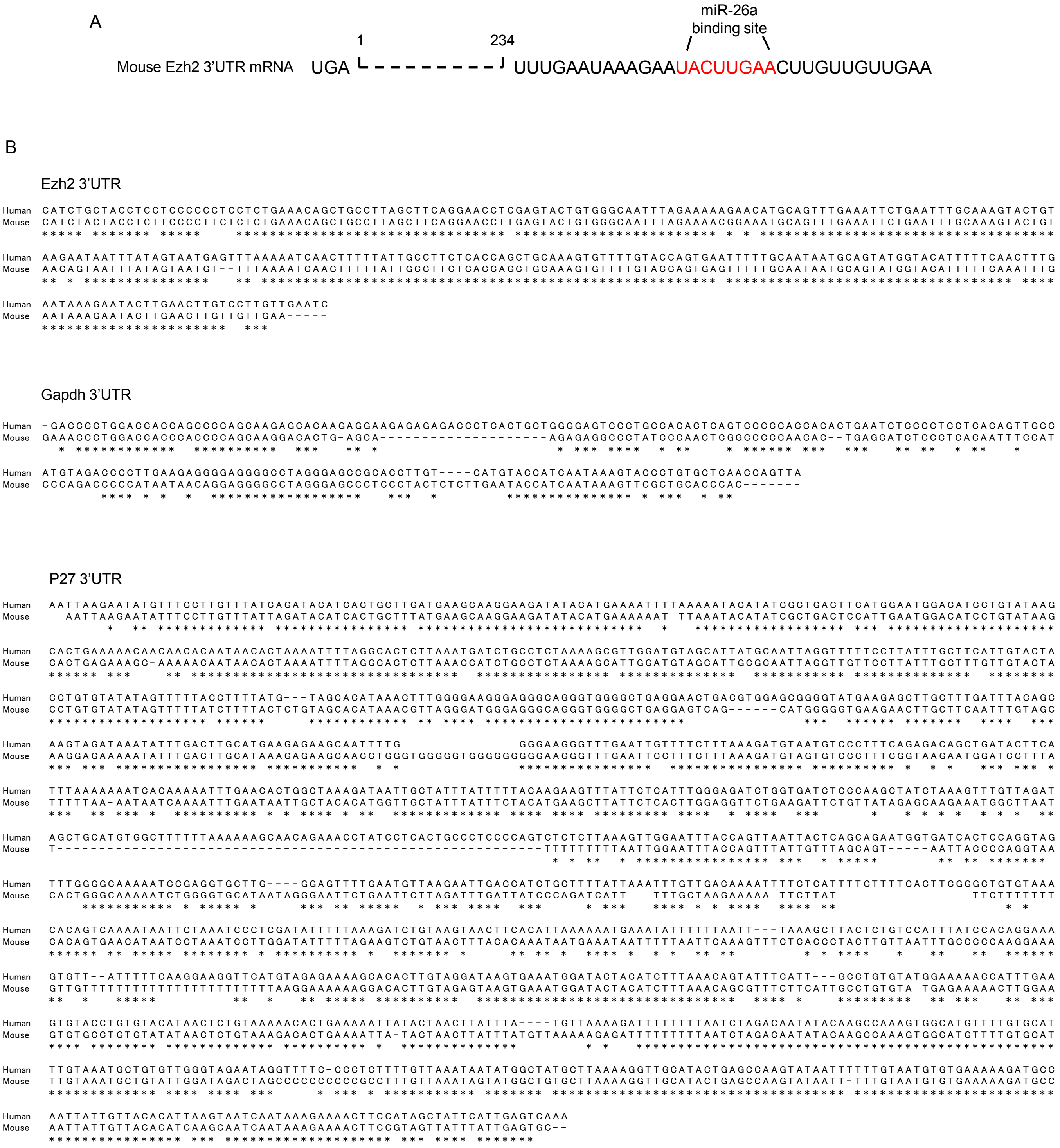


Fig. S9

Structure of the Ezh2 3'-UTR.

(A) The binding sequence of miR-26a is highlighted in red in the 3'-UTR of the mouse *Ezh2* mRNA. (B) The 3'-UTRs of *Ezh2, Gapdh,* and *p27* in human and mouse are highly conserved. Position 1 shows the base next to the stop codon.
